# Drivers and impacts of the most extreme marine heatwaves events

**DOI:** 10.1038/s41598-020-75445-3

**Published:** 2020-11-09

**Authors:** Alex Sen Gupta, Mads Thomsen, Jessica A. Benthuysen, Alistair J. Hobday, Eric Oliver, Lisa V. Alexander, Michael T. Burrows, Markus G. Donat, Ming Feng, Neil J. Holbrook, Sarah Perkins-Kirkpatrick, Pippa J. Moore, Regina R. Rodrigues, Hillary A. Scannell, Andréa S. Taschetto, Caroline C. Ummenhofer, Thomas Wernberg, Dan A. Smale

**Affiliations:** 1 Climate Change Research Centre, the University of New South Wales, Sydney, 2052 Australia; 2 Australian Research Council Centre of Excellence for Climate Extremes, The University of New South Wales, Sydney, 2052 Australia; 3 School of Biological Sciences, University of Canterbury, Private Bag 4800, Christchurch, New Zealand; 4 Australian Institute of Marine Science, Indian Ocean Marine Research Centre, Crawley, WA Australia; 5 CSIRO Oceans and Atmosphere, Hobart, TAS Australia; 6 Department of Oceanography, Dalhousie University, Halifax, NS B3H 4R2 Canada; 7 Scottish Association for Marine Science, Scottish Marine Institute, Oban, Argyll PA37 1QA Scotland, UK; 8 Barcelona Supercomputing Center, Barcelona, Spain; 9 CSIRO Oceans and Atmosphere, Indian Ocean Marine Research Centre, Crawley, WA Australia; 10 Institute for Marine and Antarctic Studies, University of Tasmania, Hobart, TAS Australia; 11 Australian Research Council Centre of Excellence for Climate Extremes, University of Tasmania, Hobart, TAS Australia; 12 Institute of Biological, Environmental and Rural Sciences, Aberystwyth University, Aberystwyth, SY23 3DA UK; 13 Department of Oceanography, Federal University of Santa Catarina, Florianópolis, Santa Catarina Brazil; 14 School of Oceanography, University of Washington, Seattle, WA USA; 15 Department of Physical Oceanography, Woods Hole Oceanographic Institution, Woods Hole, MA 02543 USA; 16 UWA Oceans Institute and School of Biological Sciences, The University of Western Australia, Crawley, WA Australia; 17 Marine Biological Association of the United Kingdom, The Laboratory, Citadel Hill, Plymouth, PL1 2PB UK

**Keywords:** Marine biology, Physical oceanography, Climate sciences

## Abstract

Prolonged high-temperature extreme events in the ocean, marine heatwaves, can have severe and long-lasting impacts on marine ecosystems, fisheries and associated services. This study applies a marine heatwave framework to analyse a global sea surface temperature product and identify the most extreme events, based on their intensity, duration and spatial extent. Many of these events have yet to be described in terms of their physical attributes, generation mechanisms, or ecological impacts. Our synthesis identifies commonalities between marine heatwave characteristics and seasonality, links to the El Niño-Southern Oscillation, triggering processes and impacts on ocean productivity. The most intense events preferentially occur in summer, when climatological oceanic mixed layers are shallow and winds are weak, but at a time preceding climatological maximum sea surface temperatures. Most subtropical extreme marine heatwaves were triggered by persistent atmospheric high-pressure systems and anomalously weak wind speeds, associated with increased insolation, and reduced ocean heat losses. Furthermore, the most extreme events tended to coincide with reduced chlorophyll-*a* concentration at low and mid-latitudes. Understanding the importance of the oceanic background state, local and remote drivers and the ocean productivity response from past events are critical steps toward improving predictions of future marine heatwaves and their impacts.

## Introduction

Prolonged periods of anomalously warm ocean temperatures, *marine heatwaves* (MHWs), are a major threat to marine ecosystems and their functioning, that have resulted in devastating and long-lasting impacts marine on scales from hundreds to thousands of kilometers^1–7^. Although these extreme events are by definition rare, marine heatwaves with notable ecological impacts have been occurring more frequently in recent years^4,8–10^. Globally, over the last century, the annual number of MHW days has increased by more than 50%^11^, explained primarily by the long-term warming of the upper ocean, although changes in variability are important in certain regions^12^. Moreover, future projections indicate order of magnitude increases in the number of MHW days by the end of the century even under scenarios of strong greenhouse gas emissions mitigation^13^. Hence there is a need to better understand mechanisms responsible for MHW occurrence and their ecological impacts.

MHWs are a rapidly developing area of research. There is growing literature examining what constitutes a MHW^14,15^, the local processes that generate and maintain them^16–18^, the large-scale climate drivers that remotely trigger or modulate their likelihood^3,16,19–21^, historical changes and trends in future projections ^e.g.^ ^11,13^, and their predictability^19,22^. Quantifying MHW characteristics enables relationships to be developed between events and their impacts on marine species and ecosystems^1,7^. The most advanced area of MHW research revolves around the dynamics, prediction and impacts of the El Niño—Southern Oscillation (ENSO). ENSO events (and events linked with other climate modes) are often associated with extreme ocean temperatures that would qualify them as MHWs, although this research is generally framed in terms of interannual variability. Considerable progress has been made into understanding the underlying dynamics of ENSO, and numerical models now operationally issue seasonal forecasts of ENSO state (including intensity and duration) with considerable skill^23^. Major ENSO events have been linked to negative ecosystem impacts including mass coral bleaching events^4,8^, distributional shifts in tropical tuna^24^ and other fisheries disruptions^25^. ENSO events and deep atmospheric convection in the tropics more generally, can also trigger remote, extra-tropical MHWs via the propagation of planetary scale oceanic or atmospheric waves^3,17,20,26,27^.

In general, the proximate causes of MHWs can be classified into three categories: (i) changes in the transport of heat by the ocean, such as boundary current intensification^2,26^; (ii) persistent large-scale atmospheric synoptic systems^17,18,20,28,29^, and (iii) coupled air-sea feedback processes such as ENSO events. The objective of this study is to identify and characterize the most extreme MHWs recorded over the satellite era using a standardized methodology, in order to explore common mechanisms/processes for their occurrence. We build a comprehensive database of extreme MHWs and perform a global analysis to determine if the events, previously described in the literature, were in fact ‘unprecedented’ and identify a large number of unstudied, yet potentially high-impact, MHWs. Unlike previous studies that have examined individual events, we systematically examine commonalities in the local processes and atmospheric conditions that initiate and terminate these events and determine regionally dependent responses of surface chlorophyll-*a* concentration to these events.

## Results

### The most intense and high-category MHW

**Figure 1:**
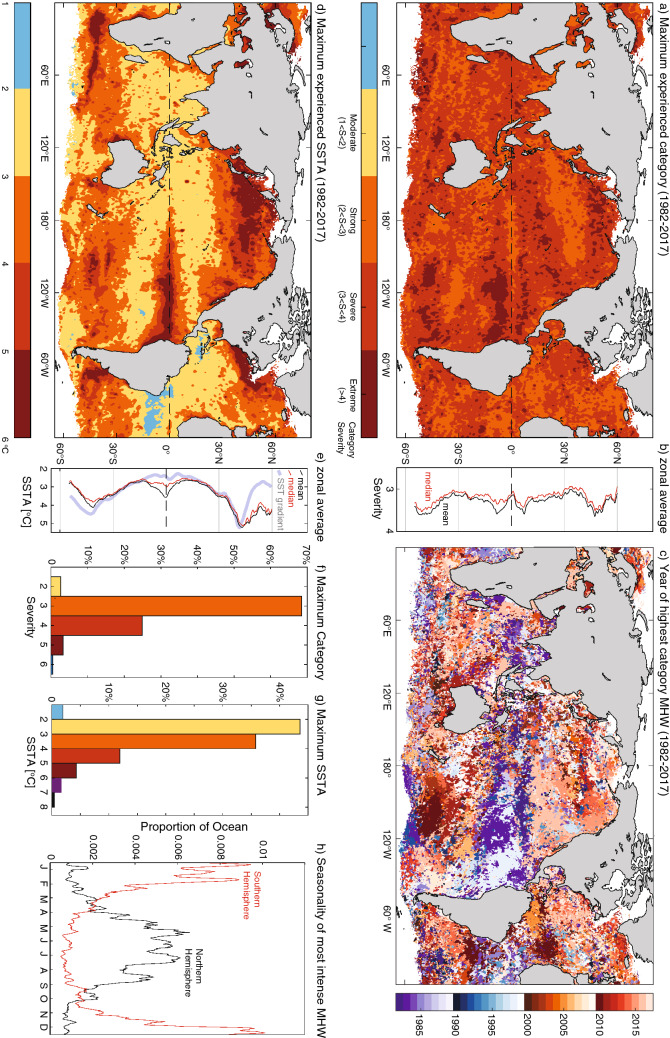
Characteristics of MHW category and intensity. **(a)** Maximum experienced category (from 1982 to 2017), **(b)** associated zonal average (red/black lines are zonal medians/means), **(f)** associated area weighted probability distributions. **(d,e,g)** as per **(a,b,f)** but for maximum recorded intensity i.e. maximum daily SSTA (in **e** grey line indicates the relative magnitude of the zonally averaged meridional SST gradient), **(c)** year of maximum recorded category (i.e. when the severity index was highest; the associated year of maximum intensity is shown in Figure S3); **(h)** proportion of ocean by month experiencing most intense MHW for northern (> 5^o^ N, black) and southern (< -5^o^ S, red) hemispheres. Colour in **(f)** and **(g)** correspond to colour bars in **(a)** and **(d)**, respectively.

Based on the MHW categorization scheme (see “Methods”)^15^, every ¼-degree grid cell across the globe experienced at least one MHW event exceeding *strong*—*category 2*—MHW conditions during the period from 1982 to 2017. Over 70% of the ocean area experienced *severe—category 3* or higher conditions, and 10% *extreme—category 4* or higher (Fig. 1a,f). As event *severity* and *category* (categories are defined from the continuous severity index, see “Methods”) depends on both sea surface temperature (SST) and on local seasonally-varying SST variability, regions with the highest recorded category MHW and highest intensity MHW (i.e. maximum SST anomalies) do not typically align (c.f. Fig. 1a,d and b,e). Regions of very high severity, for example in the eastern tropical Pacific, the Ningaloo Niño region and the south-central Pacific, indicate MHWs whose intensity were well above levels of background variability.

Over most of the ocean, maximum MHW intensities were between ~ 2.5 and  ~ 3.7 °C (interquartile range). However larger maximum intensities were evident. For example, over 5% of the ocean, the maximum recorded daily SST anomalies (SSTA) exceeded 5 °C (Fig. 1d,g). Regions of large maximum SSTA were often associated with areas of (i) strong meridional or zonal mean-state SST gradients, in particular along the Kuroshio and Gulf Stream Extensions, Agulhas Retroflection and Brazil-Malvinas confluence but also more generally in the subtropics where average meridional SST gradients are typically large (Fig. 1e); and, (ii) regions of strong poleward advection including the East Australian Current Extension and Leeuwin Current regions. MHW intensities were generally greatest at mid-latitudes. These regions were typically also associated with high temperature variability owing to displacement of fronts or large variations in heat advection. As such *severity* in these regions, which is defined relative to the local variability was not, in general, notably elevated.

In some regions, maximum intensity MHWs were clearly related to large-scale modes of climate variability. For example, the eastern tropical Pacific Ocean had high maximum SSTA, exceeding 5 ºC, associated with strong El Niño events and the suppression of upwelling, changes in surface heat fluxes and modified ocean advection. The large maximum intensity off the Angolan/Namibian coastal region was likely related to the recurring Benguela Niño events in 1984, 1995 and 2010^30^ and the 2011 maximum intensity off Western Australia was partly related to remote influences from La Niña^31^ (Fig. 1c,d).

Based on our global analysis, there was a notable seasonal difference with regards to the most intense and highest severity events. Globally, the occurrence of maximum SSTA events (and to a lesser extent maximum severity events, Figure S1) peaked in the Southern Hemisphere summer: 39% of the ocean’s surface (away from sea-ice zones) experienced its most intense MHW in austral summer (December to February; DJF), compared to 23% of the ocean’s surface in austral winter (June to August; JJA; we would expect 25% occurrence if all seasons were equally likely). Examination of the two hemispheres separately, suggested strong hemispheric enhancement of the ocean areas experiencing the most intense (and to a lesser extent most severe) MHWs during summer (Fig. 1h). In the Northern Hemisphere (north of 5^o^ N), 45% (9%) of the ocean experienced its maximum recorded intensity during summer (winter). In the Southern Hemisphere (south of 5^o^ S), the asymmetry was even more pronounced, with 62% (7%) of the ocean area experiencing events in summer (winter). We considered four factors that may influence the global and hemispheric asymmetry:

First, the area of ocean experiencing its most severe or intense MHWs was enhanced during El Niño periods, and El Niño tends to peak in austral summer (DJF). However, we found that the globally averaged seasonal asymmetry still persisted after excluding ocean areas that experienced their most intense MHW during El Niño periods (i.e. standardized Niño3.4 index > 1), albeit slightly weaker (34% in DJF, 22% in JJA). Examining the hemispheres separately, removing the effect of El Niño, widened the Northern Hemisphere seasonal asymmetry and narrowed the Southern Hemisphere asymmetry, as would be expected given El Niño typically peaks in austral summer. However, this change in seasonal asymmetry was small (Figure S1), despite the large influence of major El Niño periods (discussed below). This is because the three largest El Niño events (1982/82, 1997/98 and 2015/16), when many of the most intense MHWs occurred, were extremely long and impacted all seasons not just austral summer. As such, other factors must therefore be important.

Second, a major factor affecting the seasonal asymmetry in MHW intensities relates to shallower oceanic mixed layer depths that typically occur during the local summer season. The seasonal evolution of the most intense MHWs corresponded closely to the climatological mixed layer depth (Figure S2a,b). The enhanced intensities in summer arise as a given heat flux anomaly, from the atmosphere or from horizontal ocean advection, would generate a larger change in surface temperature when distributed over a shallower mixed layer; as such SST variability is larger, resulting in the possibility of more intense MHWs. The mixed layer depth evolution is in turn related to the seasonality in wind strength (Figure S2b), where weaker winds suppress vertical mixing thereby allowing the mixed layer to shoal^32^.

A third factor that could influence MHW seasonality is that long-term summer and autumn trends in SSTA tend to be stronger than winter and spring trends in both hemispheres^33^ (Figure S2c). However, when the seasonally varying, long-term linear ocean warming trends were removed from the SSTA timeseries, the metrics experienced negligible seasonality changes (Table S2). As such, this factor is at most a minor contributor.

Finally, in calculating SSTA intensities, a moving average was applied to the daily SST climatologies to provide a smooth timeseries (see “Methods”). This process can reduce (enhance) the climatological maximum (minimum) annual temperatures by a few tenths of a degree. As the maximum intensities occur in summer, which is a season prior to maximum SSTs (Figure S2a) this smoothing-related bias cannot explain the seasonality in intensities.

Many large contiguous areas experienced their most extreme marine heatwaves at similar times in the record. Dates for most *severe* and *intense* events were broadly consistent with each other (c.f. Fig. 1c, Figure S3) although spatial differences in SSTA variability cause some regional discrepancies. Well-documented ‘iconic’ MHWs were clearly identifiable, including the 1982/83 and 1997/98 El Niños, the 2011 *Ningaloo Niño* off Western Australia^26^, the 2009/10 Central South Pacific MHW^17^, the 2014–16 *Blob* in the northeast Pacific Ocean^3,18^ and the 2013/14 western South Atlantic MHW^20^, highlighting that these events were indeed the most intense and highest category MHWs for those regions over the satellite era.

**Figure 2:**
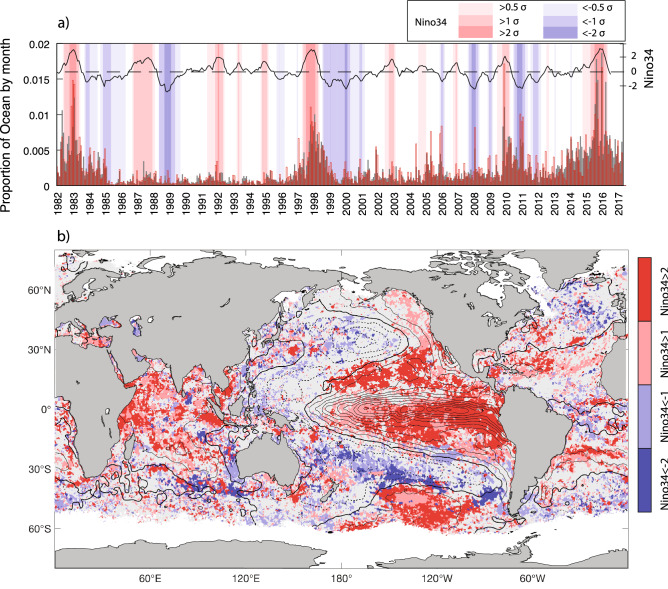
ENSO modulation of MHW severity. **(a)** Proportion of ocean area (in a given month) experiencing its most severe (grey shaded) or intense (red bars) MHWs. Shaded bands show |Niño3.4|> [0.5, 1, 2] standard deviation ($\sigma )$. Niño3.4 index and dashed zero-line superimposed (black lines); **(b)** regions of most severe MHWs that occurred when Niño3.4 > 1 (light red), Niño3.4 > 2 (red), Niño3.4 < − 1 (light blue), Niño3.4 < − 2 (blue). Superimposed regression of normalized Niño3.4 index on SSTA (interval 0.1 °C). Regression based on monthly SSTA from HadISST (1950-present). Niño3.4 represents the averaged SSTA in the region 5° S to 5° N and 170° W to 120° W.

The highest category and most intense MHWs were strongly modulated by ENSO (Fig. 2). During the three strongest El Niño events in the 35-year satellite record, ocean areas experiencing their highest category MHW rose dramatically. In particular, during the 2015/16 El Niño, in some months over 3 million km^2^ of the ocean surface experienced their highest ever category events (5 × higher than the average monthly area). Although *moderate* (standardized Niño3.4 index > 1) and *strong* (standardized Niño3.4 index > 2) El Niño conditions persisted for about 19% (6%) of the record, over 34% (19%) of the ocean surface area experienced its most severe MHW during El Niño periods. During La Niña periods, we did not find a large-scale suppression of extreme MHWs (Fig. 2a, Figure S4), as MHWs occurred in other regions. Indeed, La Niña events were often associated with an increased ocean area experiencing its highest category event. For example, during the extreme 2010/11 La Niña (Fig. 2a), extended regions in the subtropical south Pacific, Atlantic and Indian Oceans experienced their highest category MHW, including the *Ningaloo Niño* event (Fig. 1c).

During El Niño, SSTs are typically enhanced in the tropical central and eastern Pacific, northeast Pacific, the central South Pacific, the central and western Indian Ocean and parts of the subtropical Atlantic. These regions often experienced their most severe MHW during El Niño periods (Fig. 2b; red shading). However, other regions are typically associated with warming during La Niña events including the central and western subtropical Pacific, the Maritime Continent and the west coast of Australia, and these regions often experienced their most severe MHW during La Niña periods (Fig. 2b; blue shading).

Although elevated El Niño-induced temperatures are important prior to MHWs, SST preconditioning only explains a small part (a few tenths of a degree based on regression analysis) of the overall ocean temperature increase associated with extreme events which are typically 2–4 °C above climatology (Fig. 1d,g). Other coincident processes, such as persistent synoptic systems, or feedbacks that facilitate MHW intensification (e.g. wind-evaporation-SST feedback) can amplify MHWs. For example, the *Ningaloo Niño* MHW that occurred after the peak of the major 2010/11 La Niña, exhibited an ocean temperature increase well above what would typically be expected for a La Niña event of that magnitude^31^. Compounding factors included: (i) greater Leeuwin Current transport and enhanced precipitation induced low salinity^34^, (ii) unseasonably weak regional southerlies associated with local air-sea coupling, that would normally weaken the Leeuwin Current, and (iii) anomalously weak winds that suppressed turbulent ocean heat losses. Larger than expected warming was similarly noted for the extensive south central Pacific MHW in the austral summer of 2009/10^17^. In addition to the regional warming typically associated with central Pacific El Niño events, a strong and persistent high-pressure system suppressed ocean turbulent heat losses and enhanced warm southward Ekman transport^17^.

### Longest and maximum cumulative intensity MHW events

**Figure 3:**
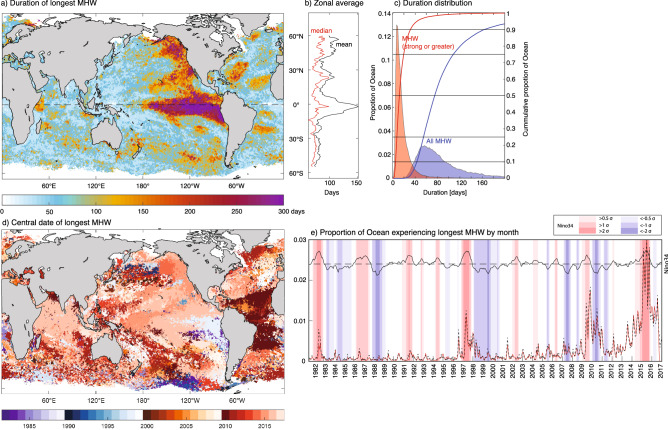
Characteristics of MHW duration. **(a)** Duration of longest recorded MHWs and associated zonal mean (black) and median (red) **(b)**. **(c)** Proportion of ocean experiencing maximum MHW duration for all MHW categories (blue) and for strong or greater MHW categories (red); coloured lines show associated cumulative totals), **(d)** central year of longest MHW; **(e)** proportion of ocean experiencing its longest (red) and largest cumulative intensity (dashed) MHW by month.

For 80% of the ocean area, the longest recorded MHWs were between 40 and 160 days (10th–90th percentile). However, across some extended regions, maximum durations were much longer (e.g. > 250 days over ~ 4% of the ocean; Fig. 3c), with the longest MHWs linked to El Niño events (Fig. 3a). The unprecedented northeast Pacific MHW in 2014–16 (the *Blob*)^18^ was also an exceptionally long event, progressing from the subtropical open ocean in 2014, to the North American coast in 2015 and then offshore again centred around 20ºN in 2016 (Fig. 3d), facilitated by extra-tropical to tropical teleconnections^3^. During the strong El Niño of 2015/16, the longest recorded MHWs occurred across wide portions of the globe, including much of the central and eastern tropical Pacific, the Tasman Sea, the northern and tropical Indian Oceans, the Maritime Continent, the south central and north-eastern Pacific and the north-western Atlantic (centred at ~ 30º N; Fig. 3d). As with MHW intensity and MHW category, we found an overall increase in the ocean area experiencing its longest MHW during El Niño periods, in particular, an unprecedented increase in the area of longest recorded events during the 2015/16 period (Fig. 3e).

In addition to strong interannual variability there was a multi-decadal increase in extreme MHW duration over the course of the record (Fig. 3e, even with the large increase in the most recent years excluded). For example, 60% of the ocean area experienced its longest duration MHW since 2010 (i.e. over the last 20% of the satellite record). This is consistent with trends in MHWs more generally^11^ and the long-term warming of the global oceans^12^. While the recent uptick in duration post 2010 may relate to internal variability (in particular, the El Niño conditions in 2010, 2014/15 and 2015/16), we note that even when background temperatures increase linearly, the duration of MHW increase nonlinearly, with duration lengthening faster as background temperatures increase. This is an intrinsic feature of the duration metric and could explain small part of the recent increase (Figure S5).

The longest duration MHWs corresponded closely with MHWs with the largest cumulative intensities (c.f. Figure 3 and Figure S6). The relationship between duration and cumulative intensity is non-linear, with cumulative intensity increasing faster with duration for more extreme events. (Figure S7). This indicates that longer events also tend to be associated with higher mean SSTA. Cumulative intensity may be a more suitable metric to understand the impacts of MHWs on marine populations that are particularly sensitive to accumulated stress. Indeed, cumulative intensity metrics like degree heating weeks are useful indicators of coral bleaching and mortality^4,35^.

### Largest MHW events

**Figure 4:**
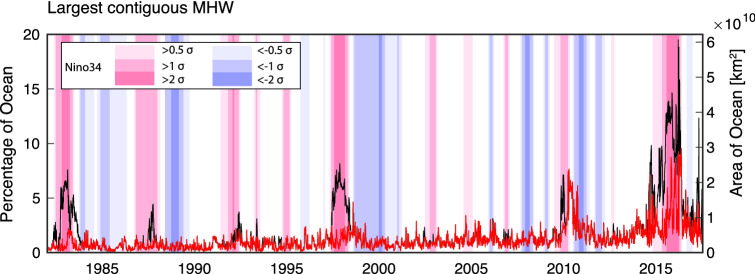
Largest single contiguous MHW each day (black lines). Red lines indicate contiguous MHW that do not intersect the equatorial central or eastern Pacific (i.e. > 170^o^ E within 5° of equator).

The largest contiguous areas (i.e. single connected event) experiencing MHW conditions occurred during strong El Niño periods (Fig. 4). In particular, during the 2015/16 El Niño, the central tropical and eastern Pacific (including the later stages of the *Blob*) experienced MHW conditions over an area exceeding 10% of the total ocean area for more than 200 days. The largest contiguous area experiencing MHW conditions (20% of the ocean surface) occurred in March 2016, across the Indian and Pacific Oceans (Figure S8). However, this unprecedented extent only persisted for a single day and was associated with the merger of multiple co-occurring MHWs (including the tropical Pacific warming associated with the El Niño, the *Blob*^3^, and the longest and strongest MHW recorded in the southeast tropical Indian Ocean^8^). This highlights a limitation of the pointwise MHW definition used here for examining spatial extent, i.e. although at each location MHW conditions must persist for at least 5 days, a MHW’s shape and spatial extent can vary considerably on daily timescale. The largest non-El Niño MHW event occurred in May 2016 across the Indian Ocean (Figure S8b). While the preceding extreme 2015/16 El Niño had dissipated by this time, this event was still likely a result of the lingering Indian Ocean Basin Warming that was a forced response to the preceding El Niño^36^. During the three strongest El Niño events, contiguous *strong* or higher category MHWs extended over 3% of the ocean surface (Figure S9). Overall, there was an upward multi-decadal trend in the spatial extent of the largest individual MHW events, which persists even if the El Niño periods are excluded (Fig. 4).

### Most extreme extremes

**Figure 5:**
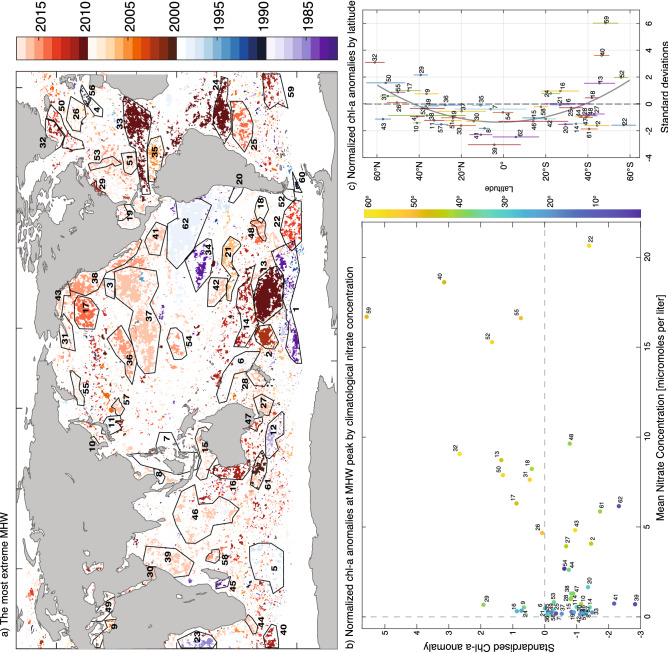
Most extreme MHW events. **(a)** Year of most severe MHW where it intersects in time with the MHW with the largest cumulative intensity (which generally corresponds with the longest MHW). Semi-contiguous regions where timing of most severe/largest cumulative intensity are similar have been manually identified (black polygons; regions 1–62). Characteristics of these regions are described in Table 1. **(b)** Normalized chlorophyll-*a* anomalies versus climatological nitrate concentration for subset of regions for which chlorophyll-*a* data is. Chlorophyll-*a* is averaged over a 24-day period centred on the MHW peak (chlorophyll-*a* anomalies are averaged over each region in **(a)** and divided by the standard deviation of the associated chlorophyll-*a* anomalies in those regions). Colours indicate the latitude (north or south of the equator). **(c)** Associated normalized chlorophyll-*a* anomalies by latitude.

The ‘most extreme extremes’ represent the largest, longest and most intense events. We defined these events where the largest cumulative intensity and maximum severity events overlapped in time over extensive regions (Fig. 5a; large coherent most extreme events are delineated by polygon regions and given an identifying number). The largest cumulative intensity events were generally also the longest (Figure S7). For each region, we determined the period when the core of the event took place (see “Methods”). Metrics associated with the top 30 most extreme events are shown in Table 1 (see Table S1 for all regions).

**Table 1:** Thirty most extreme MHWs (an expanded table showing all 62 regions is included in Table S1).

	Region	Max. intensity S > 2 °C M km^2^	Max. intensity S > 1 °C M km^2^	Max. area S > 2 M km^2^	Max. area S > 1 M km^2^	Duration IQR	Duration median days	Core date range	
1*	62	**38:** **25/11/97**	60: *6/11/97*	**11.7:** **7/11/97**	26: 6/11/97	245–317	**283**	22/6/97–14/3/98	
2	34	**31.1:** **22/12/82**	51.9: *24/12/82*	**10.3:** **22/12/82**	24.2: 8/2/83	75–96	85	13/12/82–2/3/83	
3	41	**29:** **18/11/15**	**85.3:** **2/11/15**	**10.3:** **24/11/15**	46.7: *2/11/15*	109–231	175	17/10/15–10/2/16	
4*	37	**27.9:** **12/10/15**	85.3: *2/11/15*	**11.2:** **12/10/15**	46.7: *2/11/15*	106–163	139	28/6/15–8/11/15	
5*	13	**14:** **24/12/09**	21.2: 5/12/09	4: *9/12/09*	8.7: 11/12/09	76–94	84	6/11/09–13/1/10	H
6*	38	10.5: *6/2/15*	47.4: 16/4/15	5: *6/2/15*	31.1: 15/4/15	149–243	**198**	22/11/14–21/4/15	
7*	25	10.4: *10/2/14*	17.6: 10/2/14	3.6: *10/2/14*	7.8: 9/2/14	74–98	88	11/1/14–5/4/14	
8	14	10: *21/1/11*	21.6: 22/1/11	2.8: 21/1/11	8.6: 24/1/11	50–71	62	12/12/10–27/2/11	H
9	46	10: *3/4/16*	**86.4:** **17/3/16**	**5:** **3/4/16**	**60:** **18/3/16**	69–109	86	14/3/16–24/5/16	
10	39	9.5: *14/9/15*	38.4: 17/10/15	4.4: *14/9/15*	27.9: 17/10/15	75–146	90	18/8/15–22/11/15	
11	33	8.4: 5/5/10	33.3: 19/3/10	4: *5/5/10*	24.4: 7/4/10	99–139	121	19/2/10–15/5/10	
12	31	8.2: 9/9/16	20.7: 9/9/16	2.7: 9/9/16	8.2: 9/9/16	147–273	**195**	22/8/16–18/9/16	H
13	21	8.2: 5/1/06	18.8: 4/1/06	3.2: 5/1/06	9.7: 4/1/06	85–103	97	22/12/05–11/1/06	
14*	17	7.8: 8/1/14	18.4: 16/1/14	2.7: 8/1/14	10.5: 14/1/14	141–205	**186**	12/11/13–21/2/14	H
15	24	7.8: 24/12/09	12.9: 23/12/09	3.2: 24/12/09	7.5: 10/1/10	112–158	142	11/12/09–26/1/10	
16	5	7.6: 14/2/97	16.3: 13/2/97	2.6: 14/2/97	7: 13/2/97	43–97	56	27/1/97–9/3/97	H
17	3	6.7: 30/5/97	22.7: 30/5/97	1.8: 30/5/97	11.8: 30/5/97	87.25–111	96	5/5/97–12/6/97	
18	55	6.5: 29/7/16	20.7: 9/9/16	1.6: 29/7/16	8.2: 9/9/16	35–59	54	9/7/16–9/9/16	
19	48	6.2: 19/1/15	11.6: 19/1/15	2.3: 19/1/15	5.6: 19/1/15	75–122	92	10/1/15–6/3/15	H
20	42	6: 7/4/16	**92.9:** **6/3/16**	3.4: 7/4/16	**62.3:** **6/3/16**	69–89	75	28/2/16–24/6/16	
21	1	5.9: 1/3/83	13.7: 20/2/83	2.1: 2/3/83	7.1: 20/2/83	51–99	68	16/1/83–18/5/83	H
22*	36	5.9: 21/9/14	48: 21/9/14	3.2: 21/9/14	31.3: *19/9/14*	82–111	100	5/8/14–4/11/14	
23	18	5.8: 3/2/08	12.9: 5/2/08	2.2: 3/3/08	6.2: 5/2/08	81–121	99	22/1/08–8/3/08	
24	6	5.7: 29/4/98	15.4: 28/4/98	2.6: 29/4/98	9: 28/4/98	75–139	113	8/4/98–14/5/98	H
25	54	5.6: 25/4/15	67.9: *16/7/15*	3.1: 25/4/15	40.8: *15/7/15*	143–227	**177**	11/4/15–25/7/15	
26	61	5.5: 31/12/99	8.4: 31/12/99	1.8: 31/12/99	4.2: 16/1/00	54–76	63	14/12/99–2/2/00	H
27	15	5.1: 26/6/16	17.2: 8/7/16	2.4: 26/6/16	12.2: 8/7/16	55–84	60	2/6/16–18/8/16	
28	2	4.8: 18/1/02	18.6: 30/12/01	1.6: 18/1/02	9.2: 30/12/01	71.5–95	82	6/12/01–23/1/02	H
29	53	4.5: 5/1/16	15.3: 2/1/16	2.2: 5/1/16	8.9: 4/1/16	117–170	141	22/12/15–8/6/16	
30*	27	4.3: 31/3/16	**86.4:** **17/3/16**	1.6: 31/3/16	**60:** **18/3/16**	97–164	135	7/9/15–12/7/16	

Regions are shown in Fig. 5a. Metrics shown are (1) the maximum areal intensity over the course of the MHW (spatial integral of SSTA over area with largest contiguous MHW with severity > 2 that intersects the region) [units ^o^C M km^2^], (2) as (1) for severity > 1, (3) maximum contiguous area with severity > 2 that intersects the region [units M km^2^], (4) as (3) for severity > 1, (5) Interquartile duration of maximum cumulative intensity MHW for grid cells within the region, (6) associated median duration, (7) dates of the core MHW when intensity and area of contiguous MHW and a large fraction of the region is experiencing a MHW (severity > 2, these dates are manually selected based on procedure described in the “Methods” section). For metrics (1)–(4) the date of the maximum is also shown. Bold underlined (underlined) text denotes the most extreme five (ten) MHWs associated with metrics (1)–(4) and (6). H/L/HL (last column) indicates MHWs, whose build up is associated with a strong anomalous high/low/high–low dipole pressure systems.

Many of the most extreme events were associated with El Niño periods. For example, the most extreme event in terms of spatial extent (Table 1, column 4) and spatially integrated SSTA (Table 1, column 2) over a single contiguous region (with *strong* or greater MHW category) occurred during the 1997/98 El Niño in the eastern equatorial Pacific (Fig. 5a, region 62). The largest contiguous area experiencing MHW conditions that was *strong* or greater covered almost 12 million km^2^ in November 1997. This event was associated with the longest recorded continuous MHW duration (Table 1, columns 6, 7). Other extreme extremes associated with regions 34 and 41 (regions defined in Fig. 5a) in the eastern tropical Pacific were associated with the 1982/83 and 2015/16 El Niño events, respectively.

During the Central Pacific El Niño of 2009/10, several large extra-tropical extreme events co-occurred. From mid-2009 to early 2010, an extensive event occurred in the central South Pacific (*region 13*). This event was linked to a persistent anticyclone over the region^17^ and was followed by two events in the Atlantic that extended zonally across much of the basin at about 20º S (*region 24*) and 20º N (*region 33*).

The subsequent extreme La Niña event of 2010/11 was coincident with a number of extreme extra-tropical MHWs, including high-latitude extreme events in the southern Atlantic (*regions 40* and *59*), south of Greenland (*region 32*), the central South Pacific subtropics (*region 14*), and the *Ningaloo Niño* off Western Australia (*region 16*). While the latter MHW has been dynamically linked to the extreme La Niña^31^, causal links between La Niña and these other events have yet to be demonstrated.

Our analysis highlights the evolution of the extreme MHW that constituted the *Blob* in the Pacific*.* In late 2013, a strong and persistent high-pressure system caused this event to develop in region *17*^18^. In 2014–2016, extreme MHW conditions shifted eastwards and southwards (*regions 38, 43, 36, 37, 54*) finally merging with the 2015/16 El Niño SST signature.

Importantly, only a subset of MHWs identified in Table S1 have been examined in the literature^19^. As such, previous studies have yet to establish the local mechanisms causing these intense, prolonged, and large spatial extent, links to potentially predictable large-scale climate patterns and biological impacts.

### Common MHW forcing mechanisms

Based on the most extreme MHWs, we examined the influence of proximate atmospheric mechanisms, including the roles of high- and low-pressure systems, weakened wind speeds, and anomalous air-sea heat fluxes in the evolution of these events.

**Figure 6:**
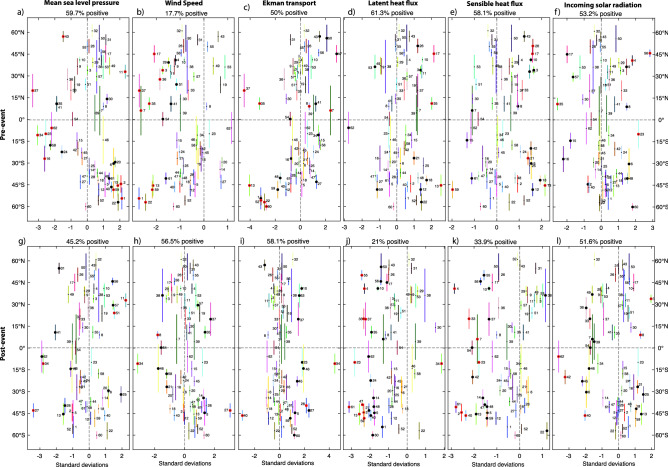
Normalised anomalies averaged over the 62 identified extreme MHW regions, before (average of 6 to 3 weeks prior to event peak, top panels) and after (average of 3 to 6 weeks after event peak, lower panels) the peak of the event. Coloured lines indicate the latitudinal extent of the MHW. Numbers indicate the regions shown in Fig. 5. Large, black circles indicate anomalies are within the top decile of anomalies for the same 4-week period across all years; large, red circles indicate the most extreme of all the anomalies for the same 4-week period across all years. Percentages above each panel indicate the percentage of regions for which anomalies are > 0.

Persistent atmospheric high-pressure systems have been indicated as a primary driver for initiating a number of prominent MHWs including the central South Pacific MHW in the austral summer of 2009/10^17^, the initial phase of the Blob from late 2013^18^, and the western South Atlantic MHW of early 2014^20^. By examining the atmospheric state prior to and during the identified extreme events, we found that a large proportion of the most extreme extra-tropical MHW events were similarly associated with large, persistent, high-pressure systems (Table 1, last column). This result is clearest in the Southern Hemisphere midlatitudes, where many of the most extreme MHW were associated with strong (top decile) or the strongest sea-level pressure anomalies (Fig. 6a). These events, which occurred in the band of prevailing westerly winds, generally formed on the equatorward flanks of the high-pressure systems in both hemispheres (e.g. Figure S10a-e). Here, the anomalous geostrophic winds would oppose the prevailing winds. Reduced wind speeds are conducive to weakened turbulent heat fluxes from the ocean and reduced vertical mixing. Furthermore, the anomalous winds would induce an anomalous poleward Ekman flow and advect warmer water into the MHW region across the mean meridional ocean temperature gradient, that tends to be strongest at subtropical latitudes. In addition, high-pressure systems are associated with subsistence and an associated reduced cloud cover. As such, increased insolation may have played an additional role in warming the near-surface ocean.

During the development of these extreme mid-latitude MHWs, we found that different subsets of the above mechanisms were operating (Fig. 6). For example, in the Southern Ocean, MHWs in *regions 1* and *59* (Fig. 5), were associated with extremely weak wind speeds and southwards (warm) Ekman transport, however surface heat fluxes were near-normal, suggesting that ocean advection and a lack of vertical mixing were important drivers for this event. Indeed, for *region 59* turbulent heat fluxes were providing a net cooling during the MHW build-up. For *regions 13 and 22*, wind speeds were again very low prior to the events. However, in these cases, latent and sensible heat losses were also very weak, suggesting that suppressed turbulent were fluxes were playing an important role for these events.

For some mid-latitude events, we found high and low-pressure anomalies dipoles straddling the MHW to its north and south (e.g. Figure S10f). Again, the anomalous meridional pressure gradient would drive anomalous easterlies that oppose the prevailing winds, potentially reducing turbulent fluxes and enhancing warm poleward advection in the surface ocean. For example, for the MHW *60* event, located south of the strong westerly winds, we found that the southward Ekman transport had its highest recorded anomaly during the event’s build up. A single mid-latitude event (*MHW 43*) was associated with the most extreme recorded low-pressure anomaly (Fig. 6a). In this case, which corresponds to the latter stages of the Blob, the MHW was located along the coast and the record-breaking low-pressure anomaly suppressed the normal upwelling of cold water along the Californian coast^3^.

In the decay phase of the mid-latitude MHWs, there is no clear bias towards anomalously high- or low-pressure systems. However, particularly in the Southern Hemisphere, the decay of the MHWs is often associated with increased wind speeds and enhanced turbulent heat flux losses. Indeed, a large proportion of extreme mid-latitude MHWs showed strong (top decile) or strongest recorded latent and sensible heat losses during the MHW decay.

In contrast to higher latitude events, many tropical extreme MHWs were associated with low-pressure systems. Some of these MHWs were again related to coastally enhanced events. In the case of the 2011 *Ningaloo Niño* (Figure S10g, region 16) the low-pressure system was associated with anomalous northerly winds along the west coast of Australia, that weakened the normal seasonal slowdown of the Leeuwin Current, maintaining anomalously strong southward transport of warm water^31^. Most other cases were related to MHWs associated with ENSO. For examples *MHWs 62, 34* and *37* in the eastern Pacific occurred during El Niño when atmospheric pressure was anomalously low over the tropical eastern Pacific.

At all latitudes, suppressed wind speeds were a common factor during the formation of a large fraction (82%) of the identified MHWs (Fig. 6b). At the same time a majority of these events (~ 60%) are associated with suppressed latent and sensible heat losses which are in turn modulated by wind speed. Indeed, across the regions there is a significant negative correlation (r = − 0.5, p < 0.1) between wind speed and latent heat fluxes. The more prevalent influence of wind speed compared to turbulent heat fluxes across the regions suggest that suppressed vertical mixing of cold subsurface waters, another processes affected by low winds, was an important driver for many of the events.

During the decay phase of MHWs, turbulent heat fluxes were clearly important for many events. 79% (67%) of extreme MHWs were associated with enhanced latent (sensible) heat losses after the peak of the event (Fig. 6j,k). Across the regions, the strengths of the latent heat flux anomalies were typically 3 × larger than sensible heat flux anomalies and almost 2 × larger than insolation anomalies. It is therefore likely that latent heat fluxes were the most prominent of the surface heat flux terms in the build-up and decay of many MHW events.

### Surface chlorophyll relationship with MHWs

In recent decades, the consequences of extreme MHWs have revealed acute and long-term impacts on marine ecosystems based on observations^1,7^. Here we consider the relationships between satellite-derived surface chlorophyll-*a* concentration (a proxy for primary productivity) and the most extreme MHWs.

Using the events identified in Table 1 and S1, a majority of the events (72%) experienced anomalously low chlorophyll-*a* during the extreme MHWs (Fig. 5b,c). This level of agreement is unlikely by chance (p < 0.01 based on a binomial test). Indeed, all the extreme tropical MHWs (between 20° N and 20° S), had suppressed chlorophyll-a levels (Fig. 5c). Interestingly, at higher latitudes the response largely changes sign with many of the extreme MHWs associated with elevated chlorophyll-*a* levels.

A recent study^37^ documented a link between MHWs in 23 coastal regions and climatological nutrients levels. Here we found a similar relationship, where regions of high climatological nitrate levels were typically associated with elevated chlorophyll-a levels at the peak of the MHW (Fig. 5b). Conversely, regions with low- to medium climatological nutrient levels were frequently associated with suppressed primary production, a suppression that was more pronounced than previously reported for coastal MHWs^37^.

## Discussion

Using a consistent framework to identify and categorize MHWs^14,15^, we identified the strongest, longest and most extensive events from the satellite era between 1982 and 2017. Given the long-term warming of most of the ocean over the last century^38^, most events are likely to be the most extreme over a much longer timeframe. By combining metrics, we provided a list of events that are both the most intense/high category and have the longest duration/highest cumulative intensity. We did not explicitly use MHW spatial extent to identify the most extreme MHWs, but most of these events extended over large areas. MHW extent can vary considerably over the course of an event and is confounded by the merging and splitting of events. An alternative approach that may be useful for understanding the importance of MHW extent on pelagic species is the concept of thermal displacement^39^, which seeks to quantify the distance a species would need to move to escape the MHW. While a small subset of the most extreme MHWs have been studied in some detail (i.e. *regions* 9, 13, 16, 17, 25, 27, 29, 36, 37, 38, 50, 62; Table 1 and S1), the local processes, large-scale drivers and regional impacts of most of these events had not been examined, prior to our analysis.

We found that the intensity, duration and extent of extreme MHWs were strongly enhanced during El Niño periods, both in the tropical Pacific and beyond. Interestingly, many extreme MHWs were also associated with the cold phase of ENSO, as La Niña events were associated with enhanced SST in some regions (Fig. 2). We also demonstrated that MHW intensity has a distinct seasonality whereby maximum intensity (and to a lesser extent severity) MHWs tend to occur in the calendar summer (of each hemisphere). We showed that the main factor in this asymmetry is likely concurrent shallow mixed layers in the local summer season that facilitate larger temperature increases for a given input of heat. Interestingly, minimum mixed-layer depths and most intense MHWs typically occur in the season prior to maximum climatological SST (Figure S2a). This mismatch may have important implications for marine species whose ecological performance depends more on the exceedance of absolute rather than relative temperature thresholds^40^. A global tendency for MHWs to occur preferentially in austral summer therefore relates in part to the larger area of ocean in the Southern Hemisphere (approximately one third greater). A compounding factor is that El Niño conditions, that are associated with more extreme MHWs, tend to peak in the December to February period.

The process of characterizing extreme MHWs allowed us to explore their commonalities. Consistent with a few previously described MHW events^17,18,20^, many of the extreme subtropical MHWs identified here were associated with anomalous high-pressure systems during their build-up. Several processes are potentially associated with high-pressure systems including subsidence that gives rise to adiabatic warming, reduced cloud cover and increased insolation and wind changes that can suppress turbulent heat losses from the ocean and vertical mixing of cold deep water to the surface and the equatorward Ekman transport of cooler waters. Our analysis suggests that different combinations of these mechanisms are important for different MHWs.

Globally we showed that almost all of the most extreme MHWs were associated with suppressed wind speeds during their build up phase. In some, but not all, cases this was also related to suppressed turbulent heat losses (in particular latent heat) from the ocean. Anomalously weak winds would also suppress vertical mixing. However, a lack of in situ subsurface data makes it difficult to quantify the importance of this latter process. While a number of extreme heatwaves were associated with extreme insolation in the build-up phase, we did not identify any systematic enhancement of solar radiation that would help MHWs form (i.e. MHWs were equally associated with enhanced and suppressed insolation). Significantly, during the decay phase of a MHW, latent, and to a lesser degree sensible, heat losses play a prominent role in cooling about 80% of the events.

Interestingly, a large majority of extreme MHWs were associated with increased downward longwave radiation both in the build-up (> 90%) and the decay phase (> 82%) of the events (Figure S11). Part of this may relate to the fact that extreme MHWs tended to cluster during periods of El Niño and during recent years, when global downward longwave radiation is elevated (Figure S11c). However, the warmer SST associated with MHWs will also generate local responses in the boundary layer that increase humidity thereby generating a positive longwave feedback, i.e. the LW change is a response rather than a driver of these large MHWs^41^.

While our analysis suggest what processes are particularly important in the formation and decay of extreme MHWs, we did not attempt to provide full heat budgets for the events. Moreover, we have only characterized the surface, not sub-surface, signature of these MHWs. Examination of subsurface processes remain a challenge^22^ given the lack of observations available to constrain mixed-layer depths, heat advection and mixing processes. While ARGO data, for example, are becoming increasingly useful, the record is short (e.g. full global coverage was not available till 2007) precluding the calculation of a reliable climatology. A possible interim solution to understanding the three-dimensional structure and processes of MHWs will be the use of ocean reanalysis. However, reanalysis products are also poorly constrained by observations in some regions and moving backwards in time^42^. Moreover, where reanalysis incorporates data assimilation, unrealistic heat fluxes are used to nudge the model towards observations, complicating heat budget analysis.

Understanding how MHWs affect ecosystems and the provision of ecological goods and services is critical for improving predictions of MHW impacts in a future warmer ocean. Only recently have attempts been made to examine general ecological responses to MHWs^7,37^. Using our database of most extreme MHWs, we examined if there are common responses in surface chlorophyll-*a* concentration, a proxy for primary productivity^43^. Processes that might link extreme ocean temperatures to changes in marine productivity include increases in vertical stratification that inhibit the upward mixing of deep nutrients or the downward mixing of phytoplankton from the photic zone or thermally driven changes in phytoplankton physiology such as altered growth and mortality rates^44^. We found that the majority of extreme MHWs are associated with reduced chlorophyll-*a* concentrations. However, the effect is dependent on latitude with strong suppression at low and mid latitudes but more frequent enhanced productivity at high latitudes. A likely explanation, is that enhanced stratification of oligotrophic tropical waters^45^, further suppresses the supply of nutrients to the upper ocean limiting growth. By comparison, at high latitudes, where phytoplankton are more often light than nutrient limited^45^, stratification reduces sinking and loss of phytoplankton out of the photic zone, thereby increasing light availability facilitating more persistent photosynthesis and blooms. Our result extends recent work that found increased chlorophyll-*a* associated with extreme summer MHWs in the polar Southern Sea, with particular strong effects in coastal zones near Antarctica^46^.

The regional differences in productivity response identified here are also consistent with a recent study^37^ that examined the surface chlorophyll response to MHWs in 23 coastal regions using the same chlorophyll dataset (but supplemented with an ocean/biogeochemical reanalysis). As in this study, they found that chlorophyll responses were strongest in regions characterized by low background nutrient concentrations. These recent analyses documenting how MHWs can affect phytoplankton productivity, are likely to have wide ecological and socio-economic importance as phytoplankton controls biogeochemical cycling^47^ and supports higher trophic levels and thereby ultimately global fisheries^48^. Future work should examine primary productivity changes on more MHWs, i.e. moving beyond the most extreme events. It will also be important to include information of vertical stratification and the effects of light availability (e.g. MHW in winter should have less impact in polar regions because of strong light limitations), to use multispectral bands to isolate more subtle community effects, and to compare results to in situ plankton data. As MHWs continue to grow stronger, larger, more frequent and longer, understanding the linkages between their occurrence and their ecological consequences, including primary productivity, will become increasing important.

## Materials and methods

Following^14^, a MHW was defined to occur when the local SST exceeded the seasonally varying 90th percentile threshold (hereafter ‘PC90’) for at least 5 days. If SST dropped below PC90 for < 2 days and subsequently returned above the threshold, the MHW was treated as a single event. We used a 30-year climatological period of 1983–2012 (inclusive) as our baseline^15^ to estimate the percentile thresholds and mean climatological SST. Code to calculate the MHW characteristics is available at https://github.com/ecjoliver/MHW_Drivers.

We used SST data from the NOAA 1/4° daily Optimum Interpolation Sea Surface Temperature v2.0^49^ (available at https://www.ncdc.noaa.gov/oisst). This is an analysis that incorporates Advanced Very High-Resolution Radiometer (AVHRR) satellite retrievals, and ship observations referenced to buoy data to account for sensor biases and differences between in situ observational platforms. We note that there have been recent concerns raised over data quality post 2016 given the deterioration of AVHRR (pers. comm. Helen Beggs, Bureau of Meteorology, Australia; 2019) and future work should test the robustness of MHW characteristics to the choice of SST products. However, given that our focus was on large extreme events that are visible in monthly SST products we expect our conclusions to be robust.

Given that PC90 presents a binary definition of a MHW, a scheme has been proposed^15^ to identify the category, or *severity*, of a MHW. For periods when MHW conditions are met, a *severity index* has been defined as:

$${S}_{i,t}=\frac{{SST}_{i,t}-{SST}_{i,d}^{clim}}{{SST}_{i,d}^{PC90}- {SST}_{i,d}^{clim}}$$

where, ${SST}_{i,d}^{clim}$ is the long-term daily mean SST on the *d*th day of the year at location *i, *${SST}_{i,d}^{PC90}$ is the PC90 SST on the *d*th day of the year at location *i*. The percentile calculation used a moving 11-day window to provide sufficient daily SST values to provide a robust PC90 estimate and a 31-day smoothing filter was applied to remove high-frequency noise (the same 31-day filter is applied when calculating the SST climatology). A MHW can therefore be categorized as *moderate* (1 < S <  = 2), *strong* (2 < S <  = 3), *severe* (3 < S <  = 4) or *extreme* (S >  = 4). This metric considers the SST anomaly relative to the local variability, making it a more applicable extreme metric for species whose thermal tolerance are adapted to local temperature variability^40^. We adopted a continuous *severity index, S*_*i,t*_, rather than simply using the discrete categories, in order to be able to rank the extreme MHWs.

Our aim was to identify the most extreme and therefore potentially also the most impactful events in the satellite era, and we therefore did not remove a secular SST trend (a step that would have been necessary if the objective was to identity strong links to climate modes^19,21^). In terms of MHW impacts this is equivalent to assuming that thermal thresholds of biological populations do not evolve following gradual global warming^50^.

MHW intensity referred to the SST anomaly relative to the local climatology (during MHW conditions) and cumulative intensity of a MHW is the integral, over time, of the SSTA for the duration of the event. The spatially largest MHWs were identified by finding contiguous (i.e. connected in any direction) grid cells experiencing MHW conditions at a given time and summing the area of these cells.

A semi-objective procedure was used to characterize the most extreme events. First, ¼ degree grid cells where the most severe recorded MHW day occurred during the largest cumulative intensity event (which also generally corresponds to the longest recorded MHW) were identified (Fig. 5a, coloured cells). Second, we manually selected regions that contained large near-contiguous areas of the most severe/largest cumulative intensity MHW occurring at almost the same time (Fig. 5a, black polygons). Third, for each of these regions, timeseries were calculated of (i) the proportion of that region experiencing moderate or strong category MHW (an example for *region 62* is shown in Figure S12a), (ii) the largest contiguous area of moderate or strong category MHW intersecting the region (e.g. Figure S12b), (iii) the integrated intensity of largest contiguous area of moderate or strong category MHW intersecting the region (e.g. Figure S12c). Fourth, the number of cells within the region experiencing their most severe recorded MHW (e.g. Figure S12d); 4. Based on these timeseries, a start and end date when most of the metrics (i–iv above) were elevated was manually identified. Finally, metrics described in Table 1 were derived based on these regions and the identified start and end dates. Use of multiple metrics provide a robust means to identify extreme MHWs that is not sensitive to a single defining MHW property.

To examine local processes important in the build-up and decay phases of the identified MHW we examine normalised anomalies of wind speed and zonal wind (at 10 m), mean sea-level pressure, downward shortwave and longwave radiation, latent and sensible heat fluxes both before (averaged over a 4 week period from 6 to 2 weeks prior to the event peak) and after (averaged over a 4 week period from 2 to 6 weeks after the event peak) the event. The standard deviation from the same 4-week period in all available years (1979–2019) at each MHW location was used to normalise the anomalies. All data were taken from the ERA-interim dataset^51^, except for downward shortwave which was from the ERA5 dataset^52^. Prior to analysis we averaged 3-hourly or hourly data to daily time series for our analysis.

Finally, to demonstrate how our global analysis of extreme MHW events can be linked retrospectively to biological impacts, we examined the response of surface chlorophyll-*a* to identified extreme MHWs. We use the gridded 8-day GlobColor chlorophyll-a product that merges multiple satellite platforms including SeaWIFS, MERIS and MODIS sensors using the GSM merging technique^53^. As the MHW data predates the merged chlorophyll-a dataset i.e. 1982 vs 1997, we were only able to examine responses for 52 of the 62 extreme MHWs in Table 1. We examined the anomalous chlorophyll-a concentration (relative to a long-term climatology) averaged over the time and area of each of the extreme MHWs normalized by the standard deviation of chlorophyll-*a* over the same region for the length of the time series. To examine whether the anomalous chlorophyll-*a* during extreme MHWs was significantly elevated, we used a Monte Carlo resampling of chlorophyll-*a* on dates that did not correspond to the extreme MHWs. A similar procedure and significance test was used to examine the distribution of normalized wind speed anomalies. Long term mean nitrate levels were extracted from the World Ocean Atlas 2013 version 2^54^.

## Supplementary information

Supplementary Information.
