# Supplementary material for: Drivers and impacts of the most extreme marine heatwaves events
Source: Sci Rep. 2020 Nov 9;10:19359. doi: 10.1038/s41598-020-75445-3 (PMC7653907; doi:10.1038/s41598-020-75445-3)
Supplement: Supplementary file 1 — Supplementary Information. [file 41598_2020_75445_MOESM1_ESM.pdf]

Supplementary Information

Drivers and Impacts of the Most Extreme Marine Heatwaves events

Alex Sen Gupta[*1,2] Mads Thomsen[3] Jessica A. Benthuysen[4], Alistair J. Hobday[5] Eric Oliver[6], Lisa V. Alexander[1,2], Michael T. Burrows[7], Markus G. Donat [2,8], Ming Feng[9] , Neil J. Holbrook[10,11], Sarah Perkins-Kirkpatrick[1,2], Pippa J. Moore[12], Regina R. Rodrigues[13], Hillary A. Scannell[14], Andréa S. Taschetto[1,2], Caroline C. Ummenhofer[15,2], Thomas Wernberg [16], Dan A. Smale[16,17]*

1. *Climate Change Research Centre, the University of New South Wales, Sydney, 2052 Australia*
2. *Australian Research Council Centre of Excellence for Climate Extremes, the University of New South Wales, Sydney, 2052 Australia*
3. *School of Biological Sciences, University of Canterbury, Private Bag 4800, Christchurch, New Zealand*
4. *Australian Institute of Marine Science, Indian Ocean Marine Research Centre, Crawley, Western Australia, Australia*
5. *CSIRO Oceans and Atmosphere, Hobart, Tasmania, Australia*
6. *Department of Oceanography, Dalhousie University, Halifax, Nova Scotia, B3H 4R2, Canada*
7. *Scottish Association for Marine Science, Scottish Marine Institute, Oban, Argyll, PA37 1QA, Scotland, UK*
8. *Barcelona Supercomputing Center, Barcelona, Spain*
9. *CSIRO Oceans and Atmosphere, Indian Ocean Marine Research Centre, Crawley, Western Australia, Australia*
10. *Institute for Marine and Antarctic Studies, University of Tasmania, Hobart, Tasmania, Australia*
11. *Australian Research Council Centre of Excellence for Climate Extremes, University of Tasmania, Hobart, Tasmania, Australia*
12. *Institute of Biological, Environmental and Rural Sciences, Aberystwyth University, Aberystwyth SY23 3DA, UK*

13. *Dept. of Oceanography, Federal University of Santa Catarina, Florianópolis, Santa Catarina, Brazil*
14. *School of Oceanography, University of Washington, Seattle, WA, USA*
15. *Department of Physical Oceanography, Woods Hole Oceanographic Institution, Woods Hole, MA 02543, USA*
16. *UWA Oceans Institute and School of Biological Sciences, The University of Western Australia, Crawley, Western Australia, Australia*
17. *Marine Biological Association of the United Kingdom, The Laboratory, Citadel Hill, Plymouth PL1 2PB, UK*

**Corresponding author: a.sengupta@unsw.edu.au*

Supplementary Table

Table S 1 Most extreme MHW. Regions are shown in Figure 5a. Metrics shown are 1) the maximum areal intensity over the course of the MHW (spatial integral of SSTA over area with largest contiguous MHW with severity>2 that intersects the region) [units ^{o}C Mkm2], 2) as 1) for severity >1, 3) Maximum contiguous area with severity>2 that intersects the region [units Mkm2], 4) as 3) for severity>1, 5) Interquartile duration of maximum cumulative intensity MHW for grid cells within the region, 6) associated median duration, 7) dates of the core MHW when intensity and area of contiguous MHW and a large fraction of the region is experiencing a MHW (severity>2, these dates are manually selected based on procedure described in the Methods section). For metrics 1)-4) the date of the maximum is also shown. Underlined (double-underlined) text denotes the most extreme five (ten) MHWs associated with metrics 1)-4) and 6). Numbers in second last column indicate references to papers detailing physical processes or biological impacts (underlined) associated with given MHWs. H/L/HL (last column) indicates MHWs, whose build up is associated with a strong anomalous high/low/high-low dipole pressure systems.

Region	Max. Intensity S>2 °C Mkm^2	Max. Intensity S>1 °C Mkm^2	Max. Area S>2 Mkm^2	Max. Area S>1 Mkm^2	Duration IQR	Duration median days	Core Date Range	Refs	
62	38: 25/11/97	*60: 6/11/97*	11.7: 7/11/97	26: 6/11/97	245-317	283	22/6/97 -14/3/98	(1, 2),(3)	
34	31.1: 22/12/82	*51.9: 24/12/82*	10.3: 22/12/82	24.2: 8/2/83	75-96	85	13/12/82 -2/3/83		
41	29: 18/11/15	85.3: 2/11/15	10.3: 24/11/15	*46.7: 2/11/15*	109-231	*175*	17/10/15 -10/2/16		
37	27.9: 12/10/15	*85.3: 2/11/15*	11.2: 12/10/15	*46.7: 2/11/15*	106-163	139	28/6/15 -8/11/15	(4)	
13	14: 24/12/09	21.2: 5/12/09	*4: 9/12/09*	8.7: 11/12/09	76-94	84	6/11/09 -13/1/10	(5)	H
38	*10.5: 6/2/15*	47.4: 16/4/15	*5: 6/2/15*	31.1: 15/4/15	149-243	198	22/11/14 -21/4/15	(4)	
25	*10.4: 10/2/14*	17.6: 10/2/14	*3.6: 10/2/14*	7.8: 9/2/14	74-98	88	11/1/14 -5/4/14	(6)	
14	*10: 21/1/11*	21.6: 22/1/11	2.8: 21/1/11	8.6: 24/1/11	50-71	62	12/12/10 -27/2/11		H
46	*10: 3/4/16*	86.4: 17/3/16	5: 3/4/16	60: 18/3/16	69-109	86	14/3/16 -24/5/16		
39	*9.5: 14/9/15*	38.4: 17/10/15	*4.4: 14/9/15*	27.9: 17/10/15	75-146	90	18/8/15 -22/11/15		
33	8.4: 5/5/10	33.3: 19/3/10	*4: 5/5/10*	24.4: 7/4/10	99-139	121	19/2/10 -15/5/10		
31	8.2: 9/9/16	20.7: 9/9/16	2.7: 9/9/16	8.2: 9/9/16	147-273	195	22/8/16 -18/9/16		H
21	8.2: 5/1/06	18.8: 4/1/06	3.2: 5/1/06	9.7: 4/1/06	85-103	97	22/12/05 -11/1/06		
17	7.8: 8/1/14	18.4: 16/1/14	2.7: 8/1/14	10.5: 14/1/14	141-205	186	12/11/13 -21/2/14	(4, 7, 8), (9)	H
24	7.8: 24/12/09	12.9: 23/12/09	3.2: 24/12/09	7.5: 10/1/10	112-158	*142*	11/12/09 -26/1/10		
5	7.6: 14/2/97	16.3: 13/2/97	2.6: 14/2/97	7: 13/2/97	43-97	56	27/1/97 -9/3/97		H
3	6.7: 30/5/97	22.7: 30/5/97	1.8: 30/5/97	11.8: 30/5/97	87.25-111	96	5/5/97 -12/6/97		
55	6.5: 29/7/16	20.7: 9/9/16	1.6: 29/7/16	8.2: 9/9/16	35-59	54	9/7/16 -9/9/16		
48	6.2: 19/1/15	11.6: 19/1/15	2.3: 19/1/15	5.6: 19/1/15	75-122	92	10/1/15 -6/3/15		H
42	6: 7/4/16	92.9: 6/3/16	3.4: 7/4/16	62.3: 6/3/16	69-89	75	28/2/16 -24/6/16		
1	5.9: 1/3/83	13.7: 20/2/83	2.1: 2/3/83	7.1: 20/2/83	51-99	68	16/1/83 -18/5/83		H
36	5.9: 21/9/14	48: 21/9/14	3.2: 21/9/14	*31.3: 19/9/14*	82-111	100	5/8/14 -4/11/14	(4)	
18	5.8:	12.9:	2.2:	6.2:	81-121	99	22/1/08		

	3/2/08	5/2/08	3/3/08	5/2/08			-8/3/08		
6	5.7: 29/4/98	15.4: 28/4/98	2.6: 29/4/98	9: 28/4/98	75-139	113	8/4/98 -14/5/98		H
54	5.6: 25/4/15	*67.9: 16/7/15*	3.1: 25/4/15	*40.8: 15/7/15*	143-227	177	11/4/15 -25/7/15		
61	5.5: 31/12/99	8.4: 31/12/99	1.8: 31/12/99	4.2: 16/1/00	54-76	63	14/12/99 -2/2/00		H
15	5.1: 26/6/16	17.2: 8/7/16	2.4: 26/6/16	12.2: 8/7/16	55-84	60	2/6/16 -18/8/16		
2	4.8: 18/1/02	18.6: 30/12/01	1.6: 18/1/02	9.2: 30/12/01	71.5-95	82	6/12/01 -23/1/02		H
53	4.5: 5/1/16	15.3: 2/1/16	2.2: 5/1/16	8.9: 4/1/16	117-170	*141*	22/12/15 -8/6/16		
27	4.3: 31/3/16	86.4: 17/3/16	1.6: 31/3/16	60: 18/3/16	97-164	135	7/9/15 -12/7/16	(10)	
28	4.3: 31/3/16	*75.5: 3/4/16*	1.6: 31/3/16	50.5: 31/3/16	118-141	127	18/3/16 -20/5/16		H
51	4: 10/9/15	15.2: 11/9/15	2.1: 10/9/15	10.2: 20/9/15	71-194	94.5	23/8/15 -8/10/15		
22	3.8: 24/1/13	9.8: 25/1/13	1.7: 24/1/13	8.1: 26/3/13	79-116	104	10/12/12 -4/4/13		H
20	3.8: 7/12/08	7: 16/12/08	1.5: 7/12/08	5.1: 16/12/08	30-35	31	30/11/08 -15/12/08		
58	3.6: 6/1/15	19.3: 27/12/14	1.5: 6/1/15	10.5: 2/1/15	110-162	*153*	23/8/14 -8/1/15		
16	3.6: 2/3/11	14: 29/3/11	0.9: 2/3/11	8.2: 29/3/11	62-108	85	4/2/11 -27/3/11	(11–13), (14–16)	L
7	3.5: 21/9/98	20.4: 26/7/98	1.6: 21/9/98	14.8: 26/7/98	43-71	51	2/7/98 -17/10/98		
59	3.5: 1/12/10	12.9: 8/2/11	1.3: 1/12/10	6.1: 8/2/11	88-140	111	26/11/10 -16/2/11		H
44	3.4: 5/1/16	9.5: 26/1/16	1.1: 5/1/16	4.8: 23/1/16	49-75	55	28/11/15 -14/2/16		
40	3.2: 10/2/11	6.5: 12/2/11	1.2: 11/2/11	3.1: 26/2/11	74-108	97	29/1/11 -8/3/11		H
23	3: 23/8/84	5.2: 23/8/84	1.6: 28/9/84	3: 26/9/84	55-81	70	9/8/84 -12/10/84		
8	3: 20/1/98	5.9: 17/1/98	1.3: 20/1/98	3.2: 18/1/98	88-100	95	11/1/98 -1/2/98		
50	2.8: 19/7/12	7.2: 14/7/12	0.8: 19/7/12	2.8: 14/7/12	48-88	79	17/6/12 -22/7/12	(17), (18)	H
35	2.5: 17/2/05	8.5: 23/3/05	1.5: 16/2/05	6.9: 22/3/05	58-74	68	20/1/05 -27/3/05		
52	2.4: 17/4/17	51.8: 18/4/17	1.2: 17/4/17	*39.3: 18/4/17*	67-100	84	1/3/17 -1/5/17		H
26	2.4: 25/11/07	7.6: 23/11/07	1.1: 25/11/07	4.3: 22/11/07	75-111	91	24/10/07 -11/12/07		H
12	2.2: 12/12/85	5.1: 21/12/85	1: 12/12/85	2.9: 21/12/85	42-51	45	21/11/85 -28/12/85		H

56	2.2: 22/7/89	7.8: 22/7/89	0.7: 22/7/89	3.8: 22/7/89	54-71	58	14/7/89 -11/8/89		H
47	2.2: 11/3/13	6.7: 12/3/13	0.8: 11/3/13	3.7: 13/3/13	68-123	82	1/3/13 -15/3/13		H
43	2.1: 18/4/16	92.9: 6/3/16	1: 18/4/16	62.3: 6/3/16	151-202	*175*	23/1/16 -24/7/16		L
11	2: 13/5/98	13.3: 14/5/98	0.6: 11/5/98	5.6: 12/5/98	51-68	61	10/4/98 -11/6/98		
32	1.8: 10/9/10	6.8: 2/9/10	0.6: 10/9/10	3.2: 26/9/10	113-159	129	20/8/10 -29/10/10		
9	1.8: 24/8/03	4.2: 24/8/03	0.6: 24/8/03	1.7: 25/8/03	58-89	83	29/7/03 -3/9/03	(19–21), (22)	
45	1.7: 22/6/83	2.6: 22/6/83	0.8: 21/6/83	1.4: 20/6/83	32-41	37	10/6/83 -30/6/83		
4	1.6: 20/11/98	6.6: 20/11/98	0.7: 18/12/98	3.8: 20/11/98	79-93	88	22/10/98 -1/1/99		
49	1.2: 16/8/15	4.3: 25/7/15	0.5: 16/8/15	2: 11/8/15	67-101	77	17/7/15 -21/8/15		
29	1.2: 22/3/12	3.1: 24/3/12	0.3: 22/3/12	1.2: 24/3/12	97-133	111.5	11/3/12 -31/3/12	(17), (18)	
19	1: 21/1/17	7.1: 5/1/17	0.5: 8/12/16	5.5: 5/1/17	87-155	116.5	28/9/16 -29/1/17		
60	1: 26/1/90	2.9: 5/2/90	0.4: 26/1/90	1.7: 6/2/90	66-79	71	4/1/90- 7/2/90		H L
10	0.9: 22/4/08	1.4: 21/4/08	0.3: 22/4/08	0.5: 21/4/08	61-109	74.5	4/3/08- 17/5/08		
57	0.7: 24/2/16	4.2: 24/2/16	0.3: 24/2/16	2.4: 11/1/16	77-106	92	20/12/15 -14/3/16		
30	0.6: 28/1/16	38.9: 3/1/16	0.4: 28/1/16	27.6: 16/1/16	74-97	78	28/11/15 -5/2/16		

Table S 2 Percentage of the ocean experienced its maximum recorded intensity in the season shown, for the global ocean, the northern hemisphere (north of 5°N) and the southern hemisphere (south of 5°S) during DJF and JJA. Percentages are provided for raw and detrended SSTA. Detrending uses a daily varying climatology of linear trends i.e. the seasonality in the SSTA trends is removed.

		Raw	*Detrended*
Global	*DJF*	*38.6*	*38.0*
	JJA	*22.3*	*22.4*
Northern Hemisphere	*DJF*	*9.0*	*9.3*
	JJA	*45.0*	*42.9*
Southern Hemisphere	*DJF*	*62.0*	*60.4*
	JJA	*6.8*	*7.8*

Supplementary figures

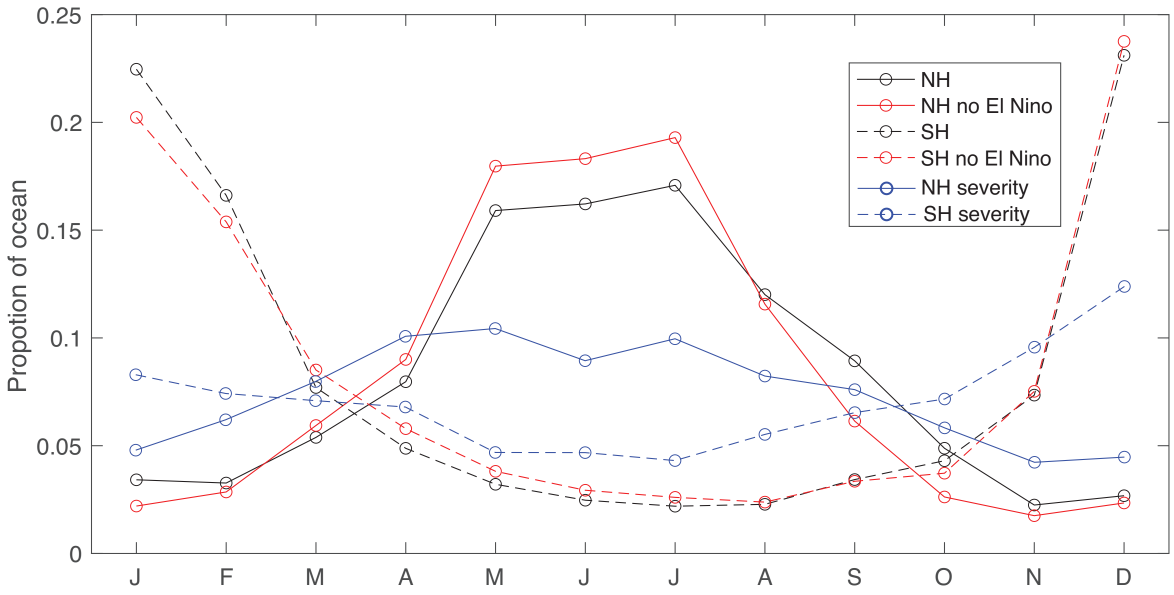

Figure S 1 Proportion of ocean experiencing its most intense MHW by month for the northern hemisphere (black solid) and southern hemisphere (black dashed). Red lines are corresponding proportions with the influence of El Nino removed (i.e. areas that experiences their most intense MHW when nino34 exceeded 1 standard deviation were excluded). Associated proportions for severity shown in blue.

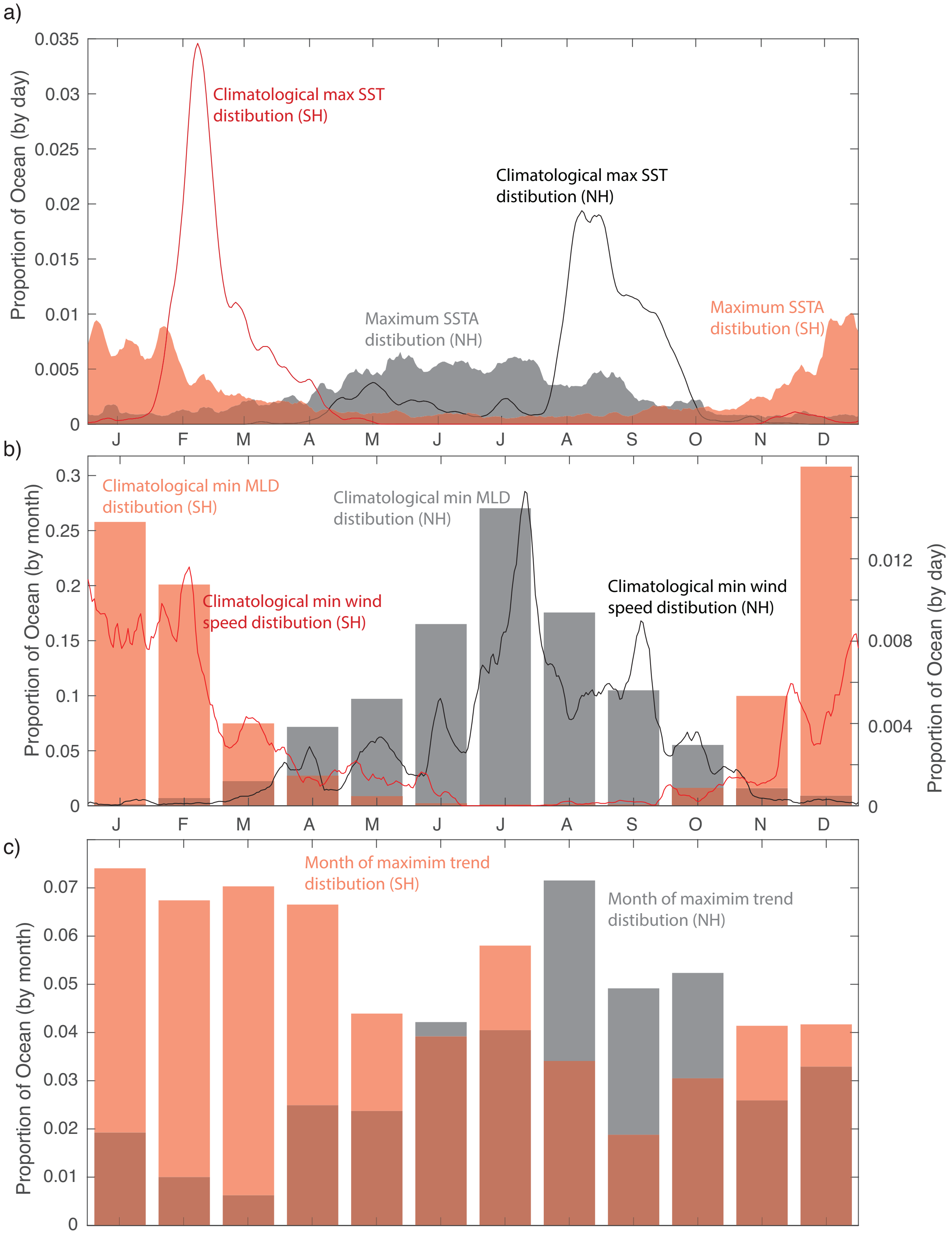

Figure S 2 Seasonal characteristics of MHWs. a) distribution (by area) of day in year when the maximum recorded SSTA (shaded) and climatological maximum SST (lines) occurred for NH (black) and SH (red); b) distribution (by area) of month of minimum climatological mixed layer depth for NH (black) and SH (red; mixed layer depth climatology from (23); c) distribution (by area) of month of maximum linear SSTA trend (1982-2016)

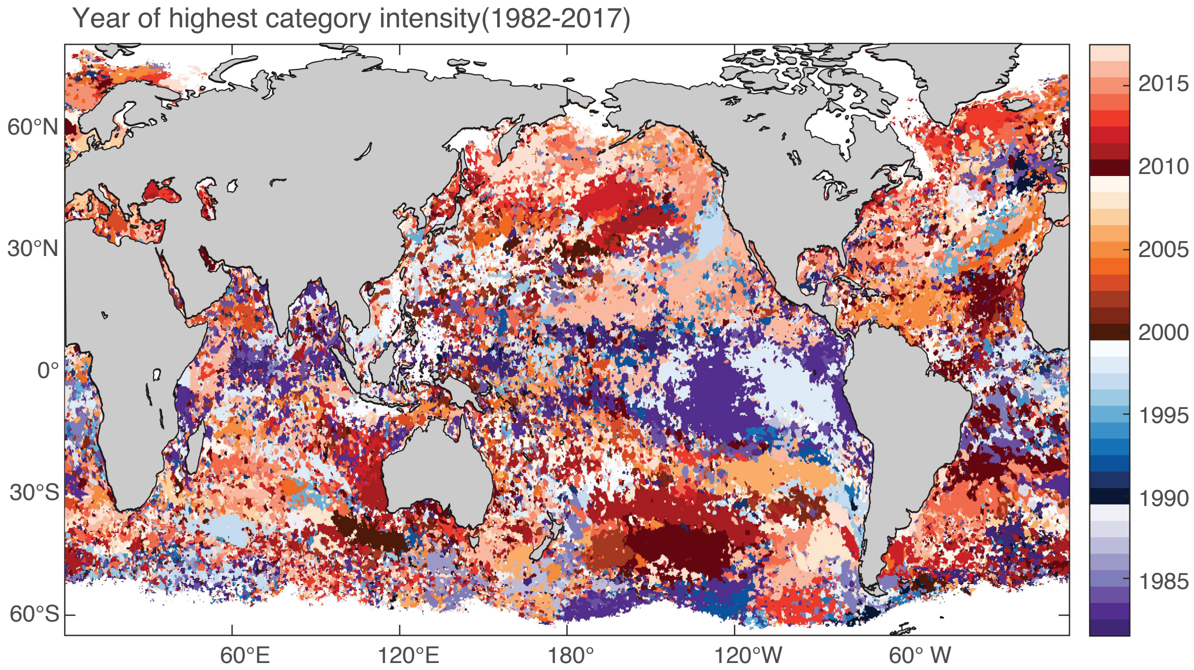

Figure S3 Year of most intense MHW (i.e. maximum SSTA during a MHW).

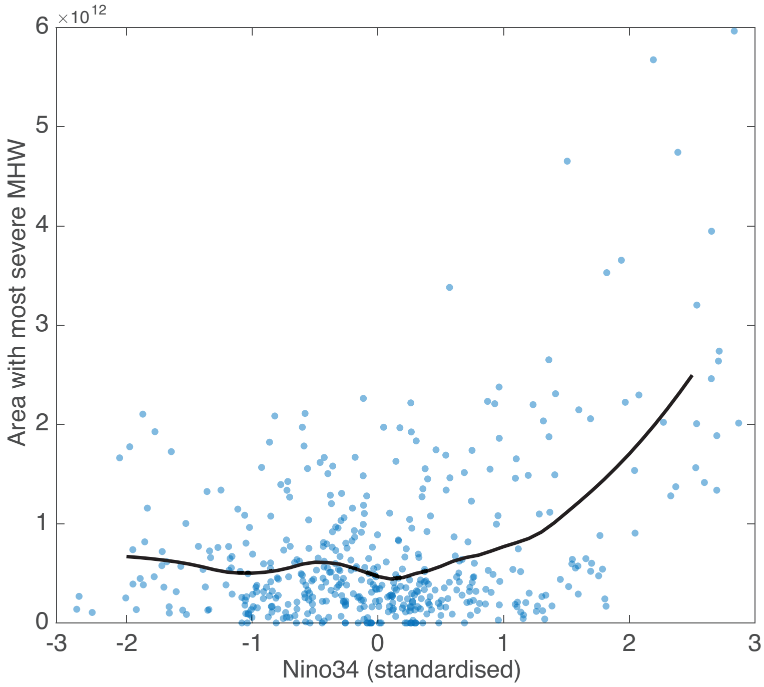

Figure S 4 Relationship between Nino34 index and area of ocean experiencing its most severe MHW. Superimposed smooth (LOWESS) fit to the data

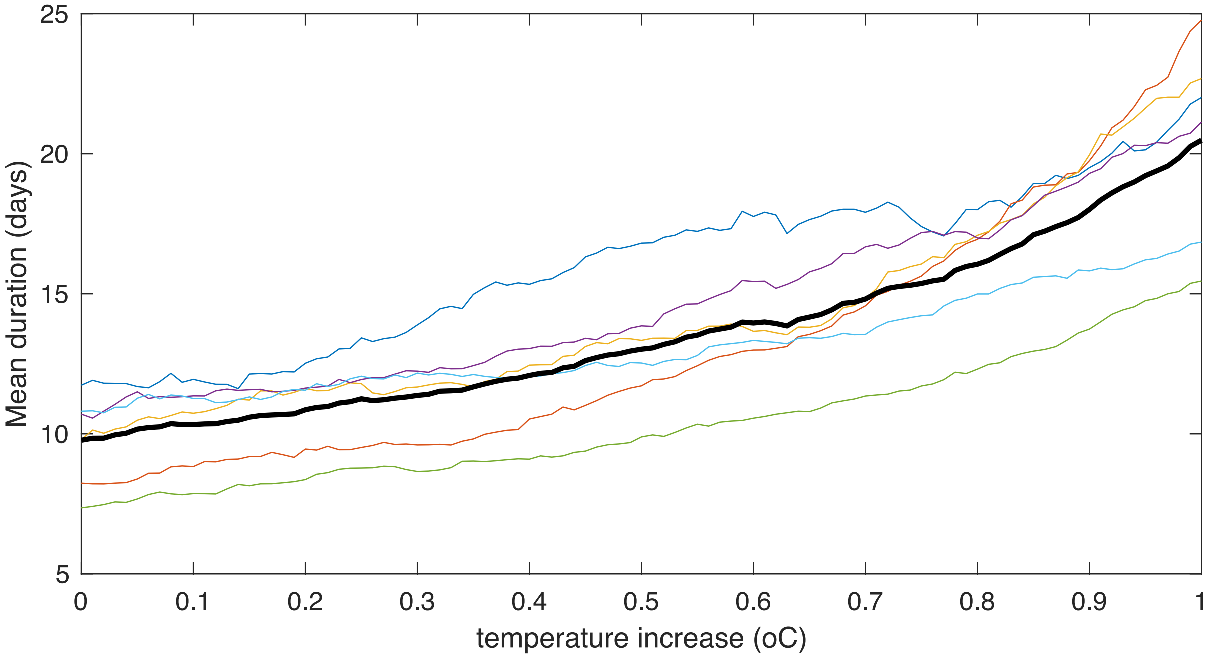

Figure S 5 Mean MHW duration as a function of background temperature increase for 6 widely separated locations (180°E, 30°S; 180°E, 10°N; 180°E, 50°N; 330°E, 30°S; 330°E, 10°N; 330°E, 50°N). At each site a 5-year high-pass filter is applied to the SSTA to remove low frequency variability and trend. The mean duration of MHWs are then calculated. This is repeated after incrementally increasing SSTA (while keeping the MHW threshold temperature constant)

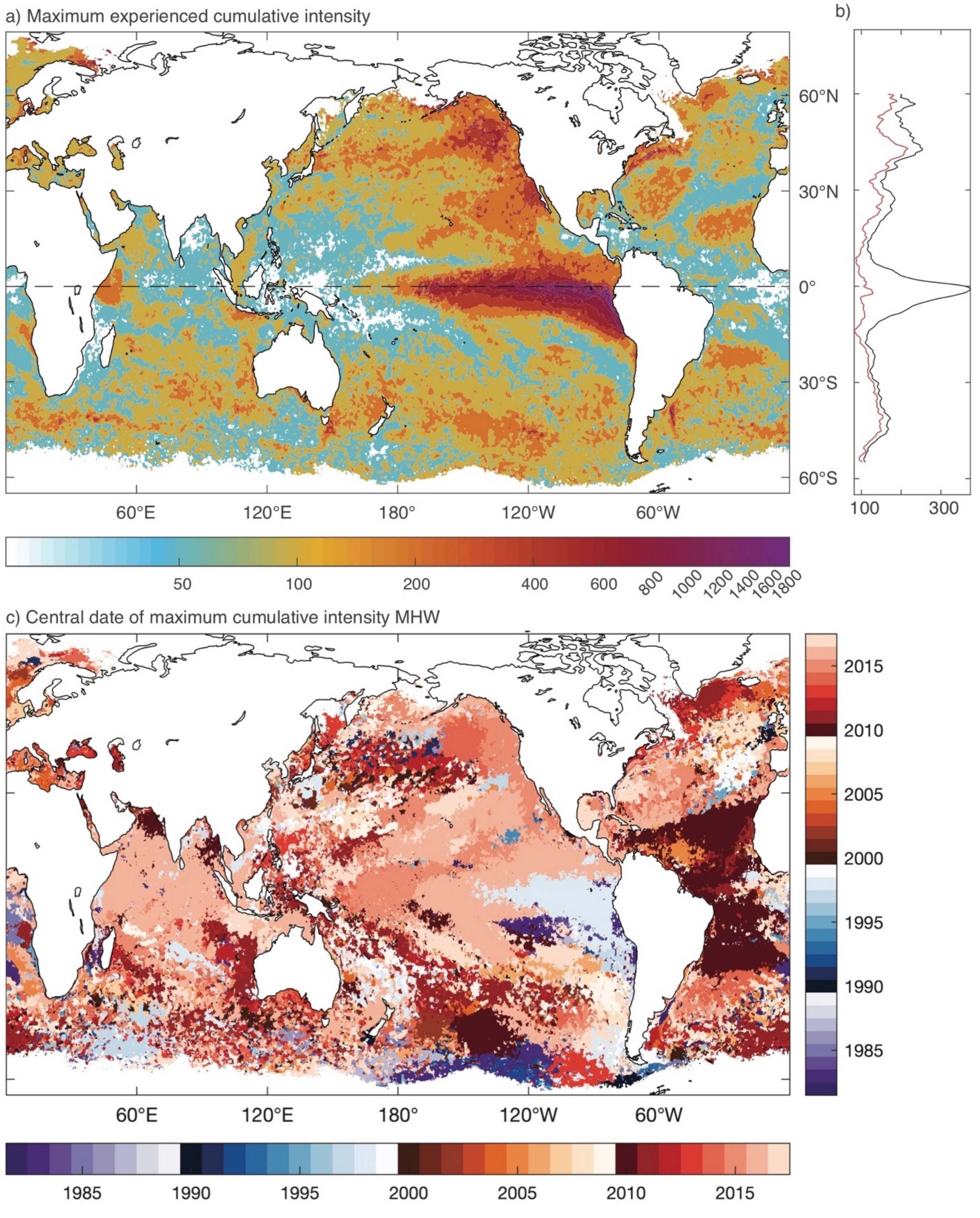

Figure S 6 Cumulative Intensity Characteristics. a) Maximum experienced cumulative intensity and the associated zonal average (b). c) central date of maximum cumulative intensity MHW.

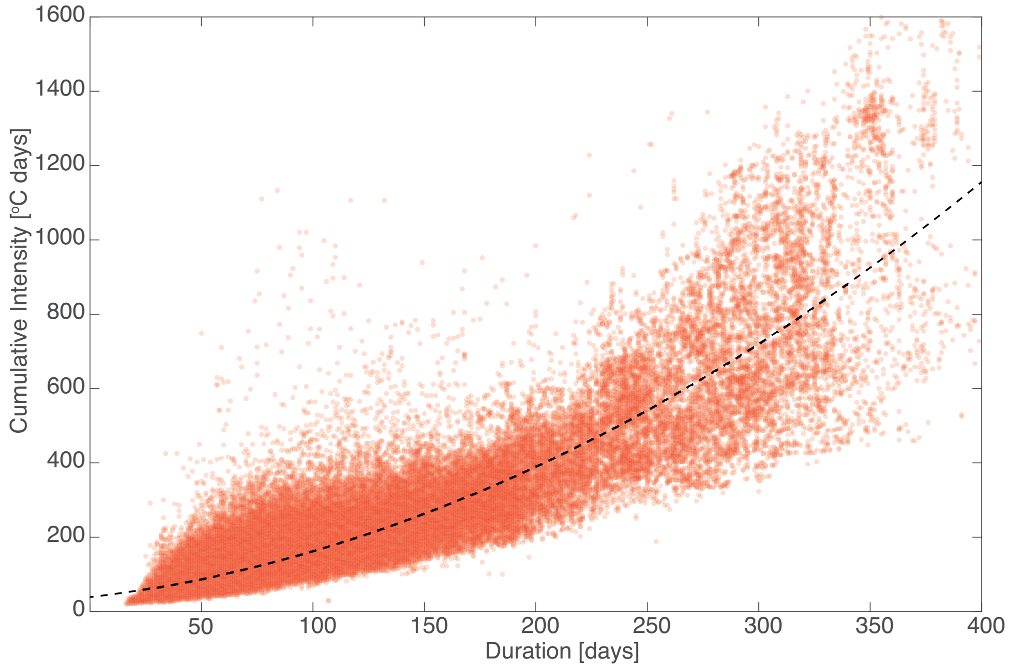

Figure S7 Maximum duration vs maximum cumulative intensity for all grid cells (best fit quadratic superimposed – dashed line)

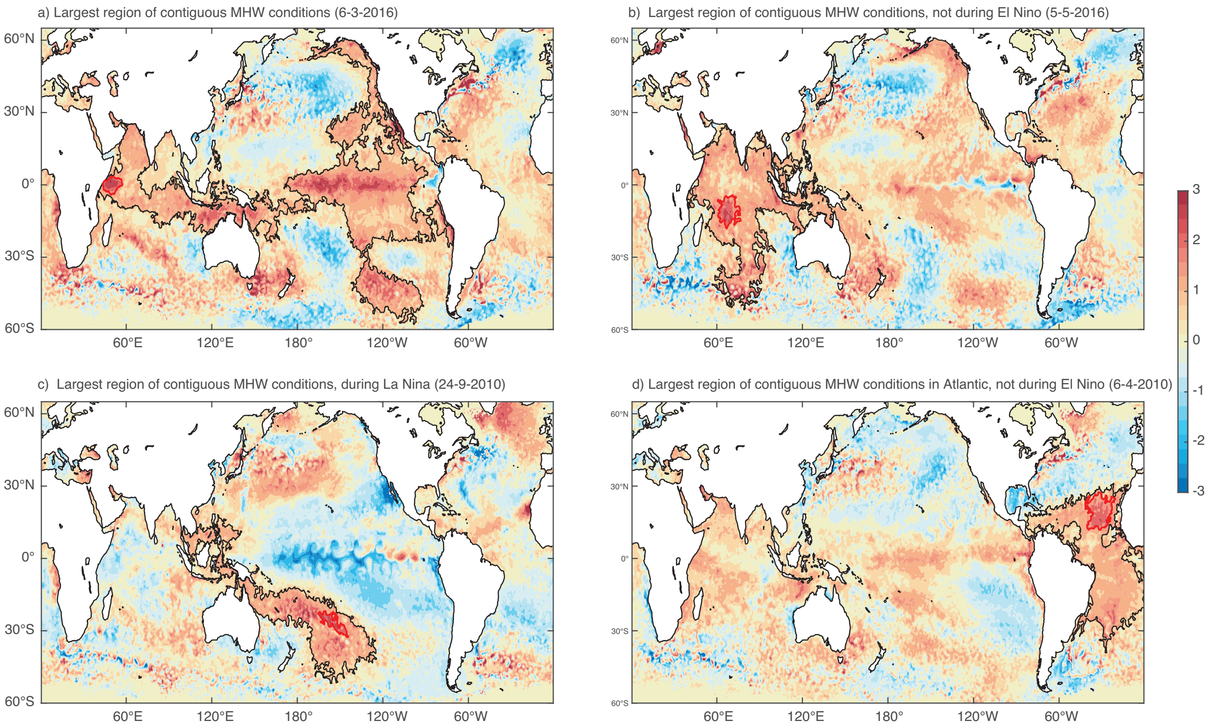

Figure S8 Largest recorded MHWs case studies. Largest contiguous regions of MHW conditions (black contours) and strong or greater MHW conditions (i.e. severity index>2; red contours)overlaying SST anomalies for four case study periods: a) largest contiguous MHW, b) largest contiguous non-El Nino MHW, c) largest contiguous MHW during La Nina and d) largest contiguous non-El Nino MHW in Atlantic

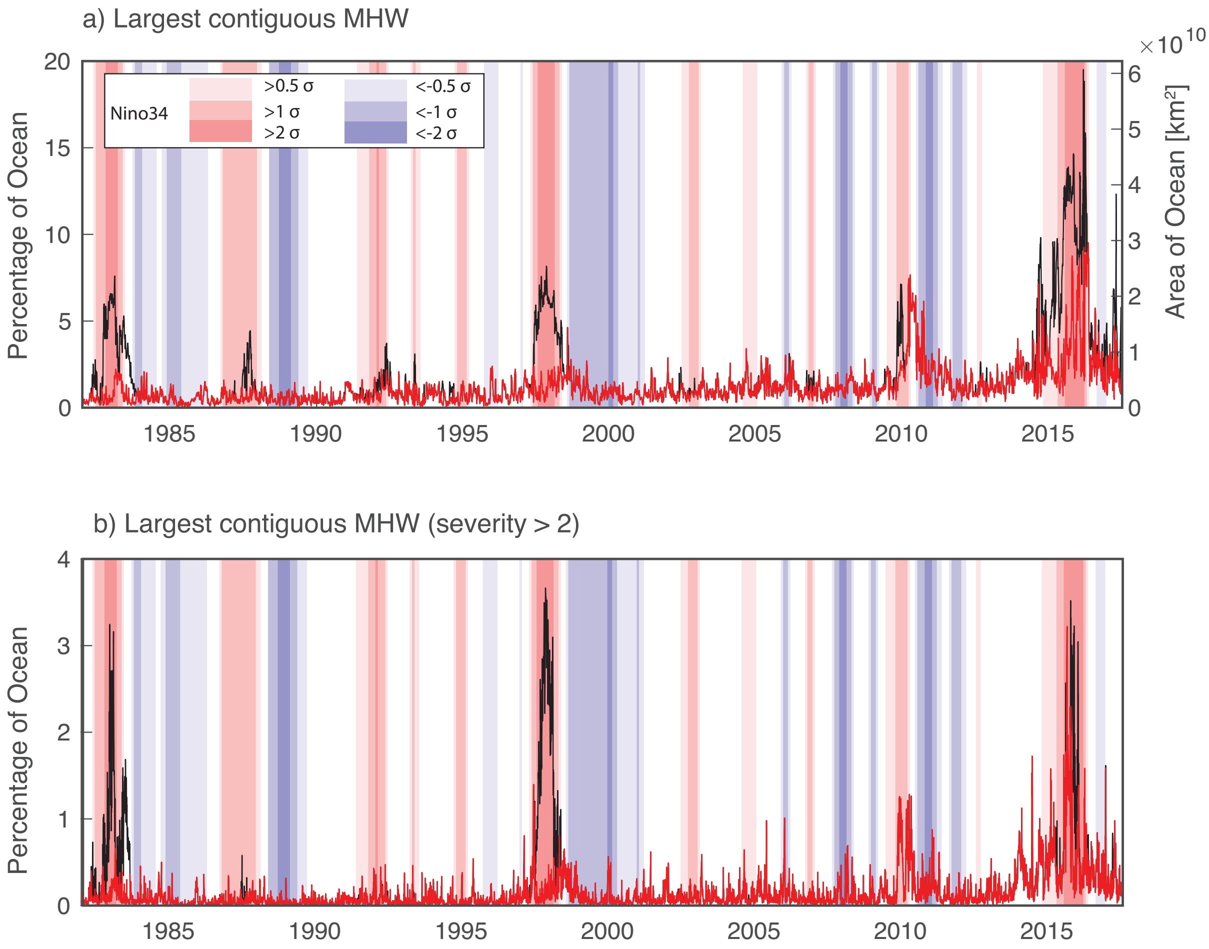

Figure S9 Largest recorded MHWs. a) Area of largest single contiguous MHW (black lines) each day. Red lines indicate contiguous MHW that do not intersect the equatorial central or eastern Pacific (i.e. >170°E within 5° of equator); b) as (a) for MHW with severity>2 (i.e. strong or greater category)

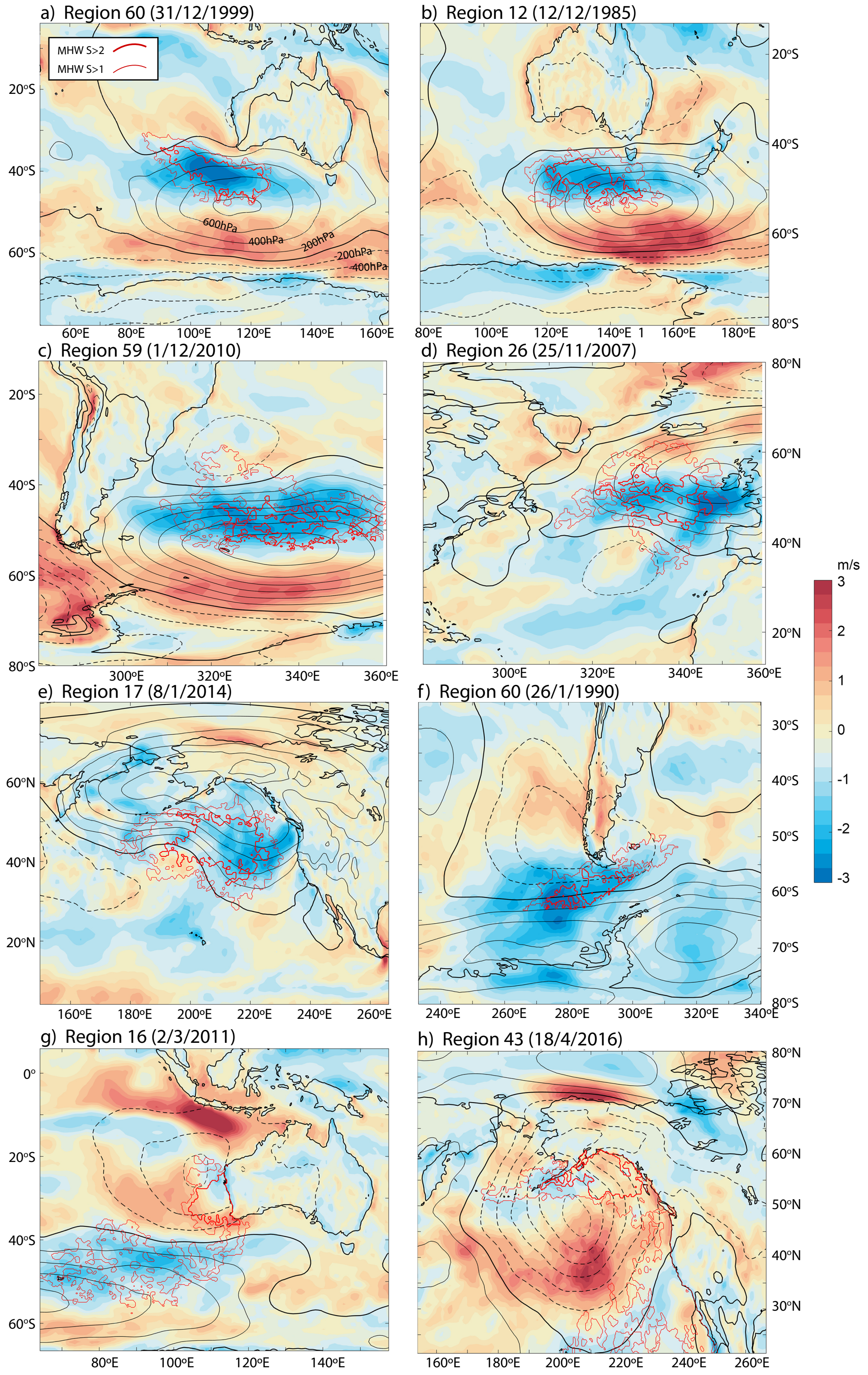

Figure S10 Synoptic conditions during a subset of extratropical extreme MHWs. Sea level pressure anomalies (black contours; interval 200hPa, positive: continuous, negative: dashed, zero: thick continuous) and wind anomalies (colours) averaged over the 60 days prior to the selected, most severe, regional MHW. Thin (thick) red lines indicate the edge of the MHW (MHW with severity>2) on the date shown. As the events selected typically have durations of 2-3 months, a 60-day window will generally correspond to the period just prior to and during the build-up of the MHW. a) December 1999, b) December 198, c) December 2010, d) November 2007, e) January 2014, f) January 1990, g) March 2011 and h) April 2016

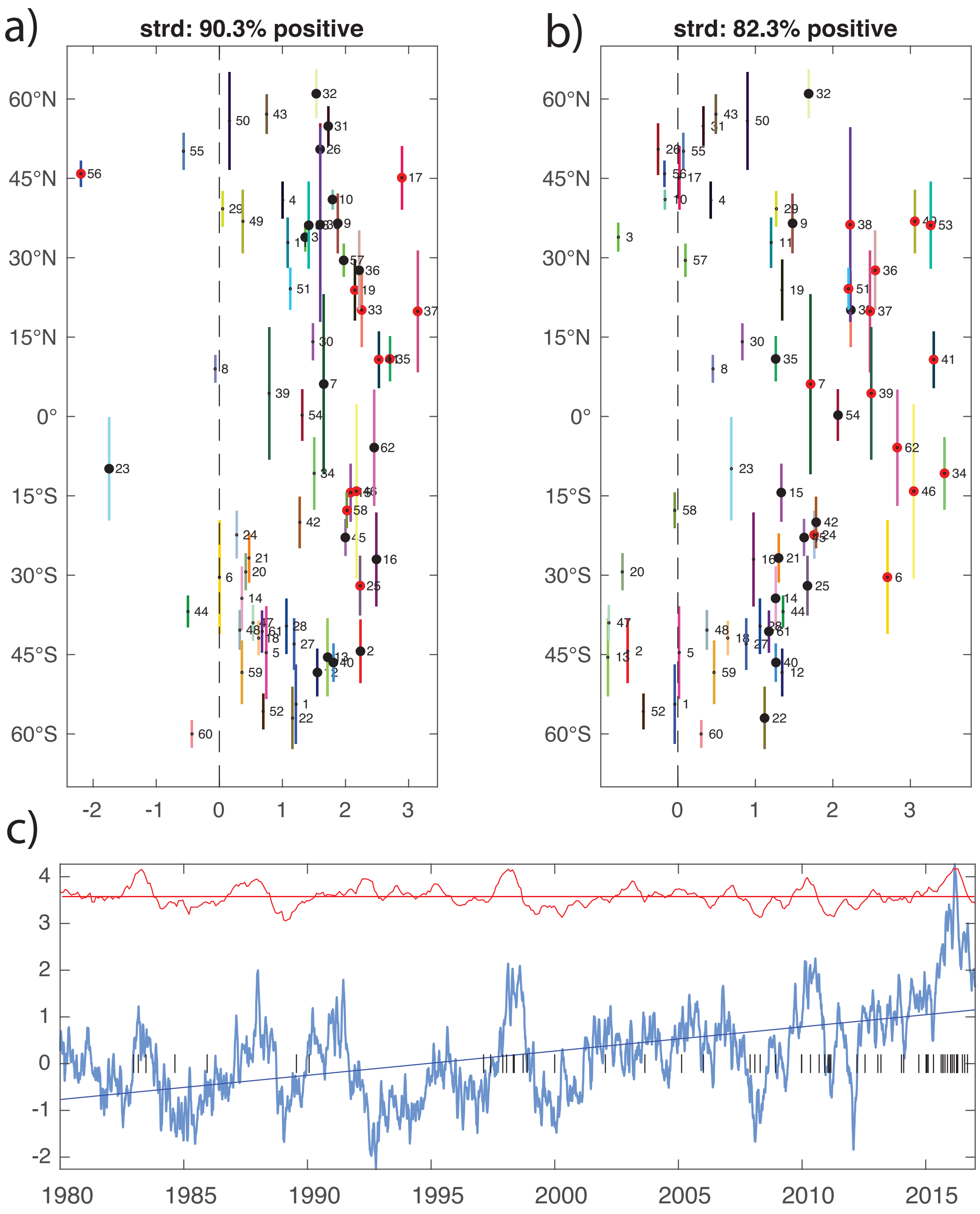

Figure S 11 Normalised anomalies of downward longwave radiation averaged over the 62 identified extreme MHW regions, a) before (average of 6 to 3 weeks prior to event peak, top panels) and b) after (average of 3 to 6 weeks after event peak, lower panels) the peak of the event. Coloured lines indicate the latitudinal extent of the MHW. Numbers indicate the regions shown in Figure 5. Large, black circles indicate anomalies are within the top decile of anomalies for the same 4-week period across all years; large, red circles indicate the most extreme of all the anomalies for the same 4-week period across all years. Percentages above each panel indicate the percentage of regions for which anomalies are >0. C). Timeseries of globally averaged downward longwave radiation (with a 4-week smoothing window) with linear trend superimposed (blue line); nino34 timeseries superimposed for reference (red line). Black vertical lines indicate timing of extreme MHWs.

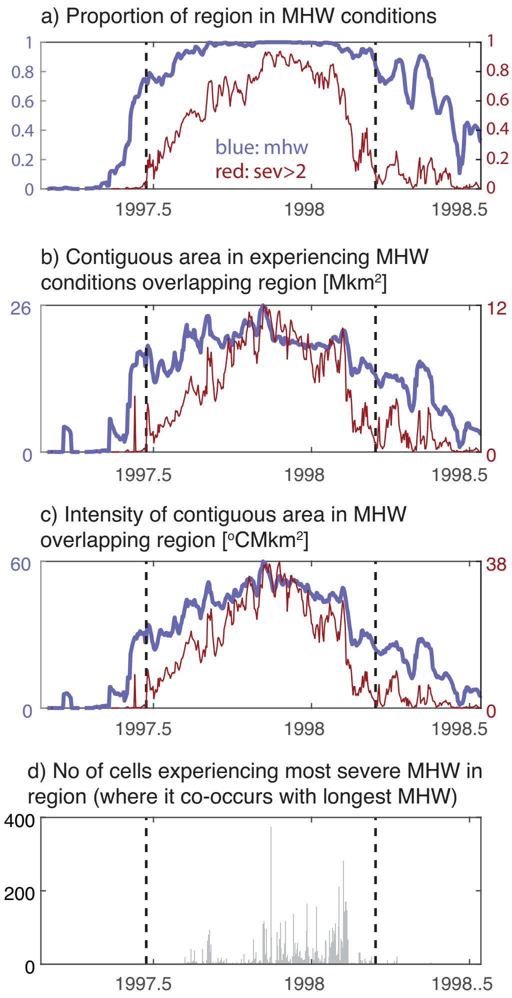

Figure S12 Selection criteria for MHW start and end dates for region 62. Daily time series of a) proportion of region, b) largest contiguous area overlapping region , c) highest intensity contiguous area overlapping region, experiencing MHW (blue) and MHW severity>2 (red). d) Daily time series of number of grid cells experiencing their most severe MHW. Vertical lines are manually identified period of core MHW, when all metrics are high.

References

1. G. Podesta, P. Glynn, The 1997-98 El Niño event in Panama and Galápagos: An update of thermal stress indices relative to coral bleaching. *Bull. Mar. Sci.* **69**, 43–59 (2001).

2. P. W. Glynn, J. L. Maté, A. C. Baker, M. O. Calderón, Coral bleaching and mortality in Panama and Ecuador during the 1997-1998 El Niño-Southern Oscillation event: Spatial/temporal patterns and comparisons with the 1982-1983 event. *Bull. Mar. Sci.* **69**, 79–109 (2001).

3. J. Picaut, E. Hackert, A. J. Busalacchi, R. Murtugudde, G. S. E. Lagerloef, Mechanisms of the 1997–1998 El Niño–La Niña, as inferred from space-based observations. *J. Geophys. Res. Oceans* **107**, 5-1-5–18.

4. E. Di Lorenzo, N. Mantua, Multi-year persistence of the 2014/15 North Pacific marine heatwave. *Nat. Clim. Change* **6**, 1042–1047 (2016).

5. T. Lee, *et al.*, Record warming in the South Pacific and western Antarctica associated with the strong central-Pacific El Niño in 2009–10. *Geophys. Res. Lett.* **37**, L19704 (2010).

6. R. R. Rodrigues, A. S. Taschetto, A. S. Gupta, G. R. Foltz, Common cause for severe droughts in South America and marine heatwaves in the South Atlantic. *Nat. Geosci.* **12**, 620 (2019).

7. N. A. Bond, M. F. Cronin, H. Freeland, N. Mantua, Causes and impacts of the 2014 warm anomaly in the NE Pacific. *Geophys. Res. Lett.* **42**, 3414–3420 (2015).

8. D. L. Hartmann, Pacific sea surface temperature and the winter of 2014. *Geophys. Res. Lett.* **42** (2015).

9. F. A. Whitney, Anomalous winter winds decrease 2014 transition zone productivity in the NE Pacific. *Geophys Res Lett* **2014**, 428–431 (2015).

10. E. C. J. Oliver, *et al.*, The unprecedented 2015/16 Tasman Sea marine heatwave. *Nat. Commun.* **8**, 16101 (2017).

11. M. Feng, M. J. McPhaden, S.-P. Xie, J. Hafner, La Niña forces unprecedented Leeuwin Current warming in 2011. *Sci. Rep.* **3** (2013).

12. J. Benthuysen, M. Feng, L. Zhong, Spatial patterns of warming off Western Australia during the 2011 Ningaloo Niño: Quantifying impacts of remote and local forcing. *Cont. Shelf Res.* **91**, 232–246 (2014).

13. A. F. Pearce, M. Feng, The rise and fall of the "marine heat wave" off Western Australia during the summer of 2010/2011. *J. Mar. Syst.* **111–112**, 139–156 (2013).

14. A. Pearce, M. Feng, W. A. D. of Fisheries, W. A. Fisheries, M. R. Laboratories, "The 'marine heat wave' off Western Australia during the summer of 2010/11" (North Beach, W.A. : Western Australian Fisheries and Marine Research Laboratories, 2011) (May 28, 2018).

15. T. Wernberg, *et al.*, An extreme climatic event alters marine ecosystem structure in a global biodiversity hotspot. *Nat. Clim. Change* **3**, 78–82 (2013).

16. T. Wernberg, *et al.*, Climate-driven regime shift of a temperate marine ecosystem. *Science* **353**, 169–172 (2016).

17. K. Chen, G. G. Gawarkiewicz, S. J. Lentz, J. M. Bane, Diagnosing the warming of the Northeastern U.S. Coastal Ocean in 2012: A linkage between the atmospheric jet stream variability and ocean response. *J. Geophys. Res. Oceans* **119**, 218–227 (2014).

18. K. E. Mills, *et al.*, Fisheries Management in a Changing Climate Lessons from the 2012 ocean Heat Wave in the Northwest Atlantic. *Oceanography* **26**, 191–195 (2013).

19. E. Black, M. Blackburn, G. Harrison, B. Hoskins, J. Methven, Factors contributing to the summer 2003 European heatwave. *Weather* **59**, 217–223.

20. A. Olita, R. Sorgente, A. Ribotti, S. Natale, S. Gaberšek, Effects of the 2003 European heatwave on the Central Mediterranean Sea surface layer: a numerical simulation. *Ocean Sci. Discuss.* **3**, 85–125 (2006).

21. S. Sparnocchia, M. E. Schiano, P. Picco, R. Bozzano, A. Cappelletti, The anomalous warming of summer 2003 in the surface layer of the Central Ligurian Sea (Western Mediterranean). *Ann. Geophys.* **24**, 443–452 (2006).

22. J. Garrabou, *et al.*, Mass mortality in Northwestern Mediterranean rocky benthic communities: Effects of the 2003 heat wave. *Glob. Change Biol.* **15**, 1090–1103 (2009).

23. C. de Boyer Montegut, G. Madec, A. S. Fischer, A. Lazar, D. Iudicone, Mixed layer depth over the global ocean: An examination of profile data and a profile-based climatology. *J Geophys Res* **109**, C12003 (2004).
